# Trionychian turtles from the Early Miocene (Burdigalian) Moghra Formation, Egypt, including a new species of Carettochelyidae

**DOI:** 10.1186/s13358-025-00358-5

**Published:** 2025-06-19

**Authors:** Yann Rollot, Mohamed K. AbdelGawad, Mohamed A. Hamdan, Ahmed N. El-Barkooky, Safiya M. Hassan, Walter G. Joyce

**Affiliations:** 1 Department of Geosciences, University of Fribourg, Fribourg, Switzerland; 2 Geology Department, Faculty of Science, Cairo University, Giza, Egypt; 3 Mineral Resources Department, Faculty of Earth Science, Beni-Suef University, Beni Suef, Egypt

**Keywords:** *Testudines*, *Trionychia*, *Carettochelyidae*, *Trionychidae*, *Trionychinae*, Anatomy, Egypt, Moghra Formation, Miocene

## Abstract

Although trionychians have a rich fossil record, much of their fossil diversity is known from the Cretaceous and Paleogene, and little is known about their evolutionary history in the Neogene. We here describe cranial and shell material of trionychians from the Early Miocene Moghra Formation of Egypt that we attribute to a new carettochelyid taxon, *Allaeochelys meylani* sp. nov., and to the *Trionyx* lineage. *Allaeochelys meylani* sp. nov. fills a temporal gap between previously described taxa and exhibits a series of unique features, including greatly thickened cranial bones, a broad bony wall posterior to the orbit, a large fossa formed by the maxilla and premaxilla at the anterior third of the triturating surface, and a medial process on peripheral II. *Allaeochelys meylani* sp. nov. also documents the oldest occurrence of *Carettochelyidae* on the Afro-Arabian continent, while the *Trionyx* material reported herein provides unambiguous evidence for the presence of this lineage on the Afro-Arabian continent no later than the Early Miocene.

## Introduction

*Trionychia* is the clade of turtles formed by *Carettochelyidae* and *Pan-Trionychidae*, two lineages of extant turtles more closely related to one another than to any other extant or extinct group of turtles (Baur, 1891; Joyce et al., 2021). Both clades are characterized by a long list of osteological features (see Joyce et al., 2021) but also the loss of carapacial and plastral scutes, which, however, occurred independently in each group. *Carettochelyidae* and *Pan-Trionychidae* notably differ from one another by exhibiting distinctive shell ornamentation patterns and by the lack of the peripherals, pygal, and suprapygal in pan-trionychids (Joyce, 2014; Vitek & Joyce, 2015). Extant trionychids are by far the more speciose of the two groups, with 35 species living across North America, Afro-Arabia, and Asia, whereas the pig-nosed turtle *Carettochelys insculpta* is the only carettochelyid remaining today, living in the northern part of Australia and southern New Guinea (TTWG, 2021).

The two clades also have a greatly contrasting fossil record. While pan-trionychids are known from dozens of species, with a particularly rich fossil record from the Late Cretaceous to Eocene of North America, Asia and Europe (Edgar et al., 2022; Georgalis & Joyce, 2017; Massonne et al., 2023; Vitek & Joyce, 2015), only 18 species of carettochelyids are currently recognized as valid from the Early Cretaceous to Miocene, with remains generally consisting of incomplete or fragmentary material (Carbot-Chanona et al., 2020; Danilov et al., 2017; Godinot et al., 2018; Havlik et al., 2014; Hutchison & Westgate, 2024; Joyce, 2014; White et al., 2023). The Afro-Arabian fossil record of both groups is particularly poor by comparison to other regions (Avrithis & Georgalis, 2024; Carbot-Chanona et al., 2020; Chroust et al., 2023; Collareta et al., 2024; Georgalis & Joyce, 2017; Joyce, 2014; Vitek & Joyce, 2015; White et al., 2023). Indeed, if Eocene to Holocene fragments are ignored, the Afro-Arabian fossil record of pan-trionychids is limited to three valid cyclanorbines from the Miocene and Pliocene of Kenya while only one valid carettochelyid is known from the Middle Miocene of Libya (Andrews, 1914; Georgalis, 2021; Georgalis & Joyce, 2017; Havlik et al., 2014; Joyce, 2014; Meylan et al., 1990).

Here, we report new trionychian material from the Early Miocene (Burdigalian) of the Moghra Formation of Egypt. The Moghra Formation previously yielded a diverse assemblage of vertebrates represented by mixture of marine, fresh water and terrestrial types, including mammals (AbdelGawad, 2011; Miller, 1999; Miller et al., 2009, 2014; Morlo et al., 2007, 2019; Pickford & AbdelGawad, 2024; Pickford et al., 2009, 2021; Rasmussen et al., 1989; Sanders & Miller, 2002), fishes (AbdelGawad et al., 2016; Priem, 1920), sharks (AbdelGawad et al., 2016; Cook et al., 2010), birds (Miller et al., 1997; Smith, 2013), crocodiles (AbdelGawad, 2016; AbdelGawad et al., 2014; Andrews, 1900; Blanckenhorn, 1901), squamates (Georgalis et al., 2020), and turtles, mostly podocnemidids (AbdelGawad, 2016; AbdelGawad et al., 2018, 2024; Dacqué, 1912; Gaffney et al., 2011; Reinach, 1903; Zonneveld et al., 2022). The formation previously also yielded numerous trionychian remains (AbdelGawad, 2016; AbdelGawad et al., 2018; Dacqué, 1912; Reinach, 1903), but this material is now recognized to be undiagnostic beyond the clade level (de Lapparent de Broin, 2000; Georgalis & Joyce, 2017). The carettochelyid remains that we describe here are sufficiently preserved to allow recognition of a new species of Carettochelyinae (i.e., *Allaeochelys*/*Carettochelys* lineage or *Allaeochelys*-node sensu Joyce, 2014) and represent the oldest known carettochelyid taxon in Afro-Arabia. The pan-trionychid remains can be identified as being part of the *Trionyx* lineage and likely represent a new species, too. We, however, refrain from naming a new taxon as the material lacks diagnostic features that would allow rigorously distinguishing it relative to other valid *Trionyx* species from the Middle–Late Miocene and Pliocene, despite a clear temporal gap (Fucini, 1912; Georgalis & Joyce, 2017; Peters, 1855).

## Geological setting

**Fig. 1:**
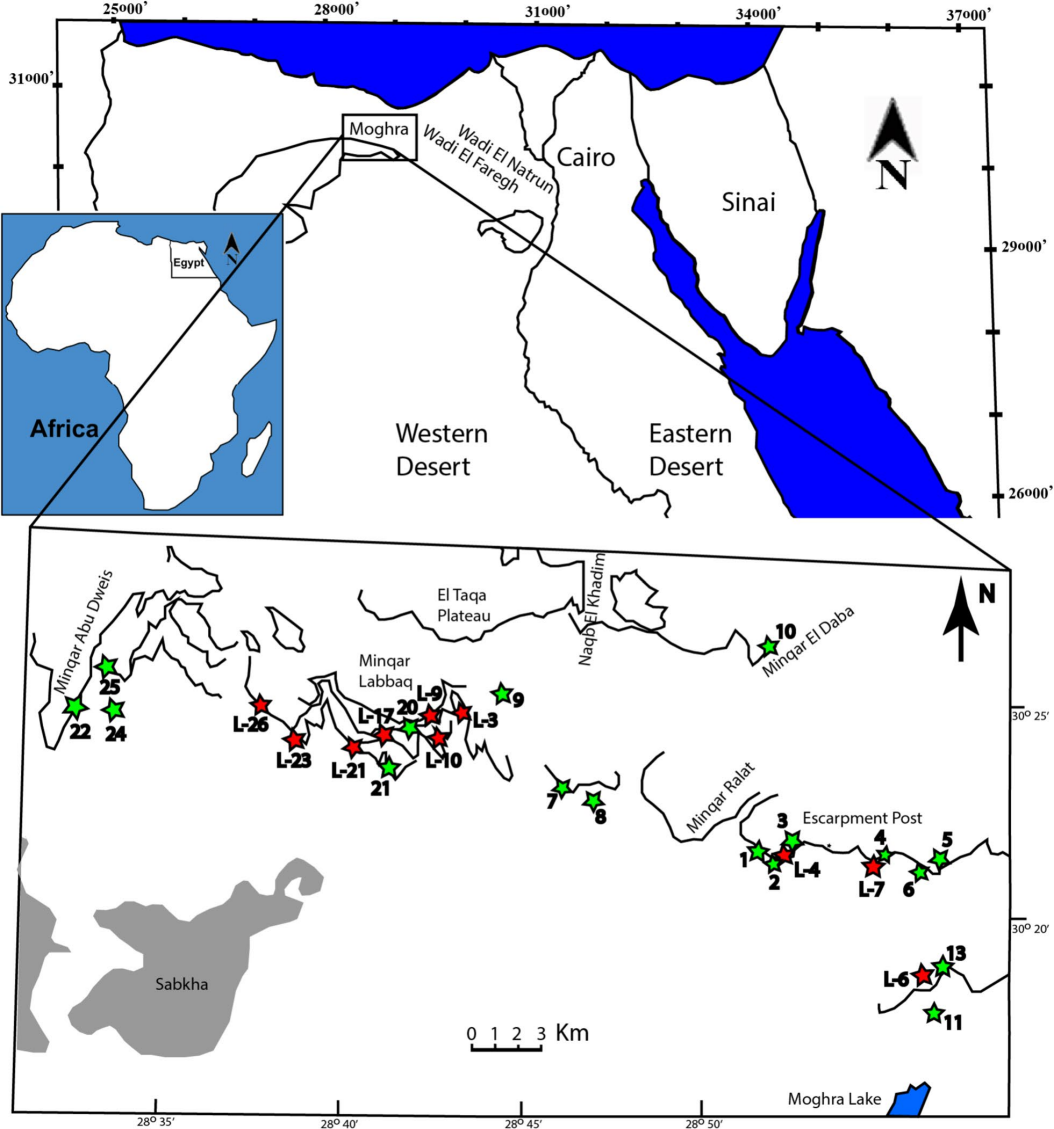
Map of Egypt indicating the location of Moghra, Wadi Faregh, and Wadi Natrun and the fossiliferous localities (quarries). Red stars indicate localities reported from twentieth century excavations, green stars indicate localities discovered by more recent prospecting and excavations (co-authors MKAG, MAH, ANEB, and SMH). Specimens described in the present study were found in the following localities: DPC 4466 at L-3; CGM 67140, DPC 3622, and DPC 4122 at L-4; DPC 12585 and DPC 14555 at L-5; DPC 12637 at L-9; CGM 67151, DPC 6436A, and DPC 6436B at L-17; DPC 7742 and DPC 7789 at L-23; DPC 7741 at L-26

The Moghra Formation, which is named after the Moghra Oasis, crops out in the northeastern part of the Qattara Depression, about 60 km south of El Alamein, Egypt (Fig. 1). The formation consists of about 400 m of siliciclastic sediments (AbdelGawad, 2011, 2016; AbdelGawad et al., 2014, 2016, 2018; Hassan, 2013; Hassan et al., 2012), overlies the Dabaa Formation, which consists of an Oligocene marine shale, and underlies the Marmarica Formation, a marine limestone from the Middle Miocene that forms the top of the Qattara escarpment (Miller, 1999; Tawadros, 2001).

The Moghra Formation was successively interpreted as fluviomarine, semicontinental, and estuarine sediments (Abdallah, 1966), fluviomarine sediments (Marzouk, 1970), or shallow marine to neritic and as restricted mixed fluviomarine (Khaleifa & Abu Zeid, 1985). More recent geological investigations suggested the Moghra Formation to represent a sandy estuarine complex that consists of a series of stratigraphic units that reflect repeated transgressive to regressive shoreline movements across the Burdigalian coastal landscape (Hassan et al., 2012). Nine transgressive–regressive units were identified, each of which is capped by a river-scour surface that severely truncates the underlying regressive half-unit (Hassan et al., 2012). The transgressive part of each unit is preserved atop a deep erosional scour surface and consists of tidal–fluvial sandstones with silicified tree trunks and vertebrate remains. These sandstones transition up to cross-stratified, tidal estuarine channel deposits and then to open-marine, shelf mudstones and limestones (Hassan et al., 2012). The regressive part, on the other hand, is thinly developed, consisting of thin-bedded, fossiliferous shelf mudstones that pass upward to thin, tide-influenced delta-front deposits (Hassan et al., 2012). Thus, the paleoenvironment of Moghra has been proposed to be a series of estuarine units stacked in a net transgressive stratigraphy (tide dominated estuary environment) (AbdelGawad, 2011; Hassan, 2013).

Vertebrate fossils were mostly recovered from four stratigraphic horizons found along the basal part of units II, VI, VIII, and X. The fossil horizons are known as F1, F2, F3, and F4, respectively (AbdelGawad, 2011, 2016; AbdelGawad et al., 2010, 2012). Each horizon represents an erosional lag surface composed of mudclasts associated with coprolites and silicified wood. The vertebrate faunas are more concentrated in the lower horizons F1 and F2. There is a decrease in the abundance and diversity of vertebrate remains associated with the decrease in the lag deposits in the younger units, which more closely resemble tidal channel deposits than in the older units (AbdelGawad, 2011).

Faunal correlations with sites in East Africa suggest that the Moghra Formation is approximately 18–17 Ma (AbdelGawad, 2011; Miller, 1999; Morlo et al., 2007). However, more recent strontium isotope (^87^Sr/^86^Sr) age-dating analyses of macrofossil fragments suggested a range from 20.5 to 17 Ma, corresponding with the Early Miocene (Burdigalian) (Hassan, 2013). Most of the fossils were collected from the lower-middle part of the section and are dated between 19.6 and 18.2 Ma, although a few specimens are derived from deposits approaching 17 Ma (Hassan, 2013).

The Wadi Faregh (El–Farigh) Depression is located in the northeastern part of the Western Desert and covers about 826 km^2^. It lies between Wadi Natrun in the east and the Moghra Depression in the West. Wadi Faregh contains the Moghra Formation, whose 150 m thickness consists mainly of coarse sands, sandstone and clay interbeds with vertebrate remains and silicified trees and is gravelly at the base. The dominant environment is fluvio-marine (Stromer, 1907; Said, 1962; Pickford & AbdelGawad, 2024) with delta plain deposits (Said, 1990). Stromer (1907) mentioned vertebrate remains such as terrestrial mammals (anthracotheres, proboscideans), marine mammals (i.e., dolphins), fishes, turtles and crocodiles from the Lower Miocene deposits at Wadi Faregh, near Wadi Natrun, Egypt. The anthracothere remains from Wadi Faregh are limited in diversity but agree with the fauna from Moghra localities and support a relationship to the Early Miocene fauna (Pickford & AbdelGawad, 2024).

## Material and methods

The carettochelyid material described herein consists of several isolated plates of the carapace, three fragments that correspond to incomplete remains of the carapace and plastron, and a partial cranium. The trionychid material consists of six pieces that altogether represent a nearly complete carapace and three elements that correspond to partial hyo- and hypoplastra. The carettochelyid and trionychid material is housed at the Duke Lemur Center Museum of Natural History (DPC) in Durham, North Carolina, USA and Egyptian Geological Museum (CGM), Cairo, Egypt (see referred material below). The carettochelyid cranium DPC 7742 was subjected to X-ray micro-computed tomography at the Shared Materials Instrumentations Facility (SMIF) of Duke University, using a Nikon Metrology XTH 225 ST scanner, with a voltage of 215 kV, a current of 451 µA, 3116 projections, an exposure time of 267 ms, and a copper filter. The scanning resulted in 1798 coronal slices with a voxel size of 97.5 µm. The dataset was segmented in Mimics Innovation Suite 25 (https://www.materialise.com/en/healthcare/mimics-innovation-suite) with the lasso and interpolation tools. The segmented bones were exported as.ply files. Blender 2.79b (https://www.blender.org) was used to visualize the segmented objects and create high-quality illustrations and figures. The µCT data and 3D models were deposited at MorphoSource (https://www.morphosource.org/projects/000694201).

## Institutional abbreviations

**BSPG**, Bayerische Staatssammlung für Paläontologie und Geologie, München, Germany; **CGM**, Egyptian Geological Museum, Cairo, Egypt;** DPC**, Duke Lemur Center Museum of Natural History, Durham, North Carolina, USA; **HLMD**, Hessisches Landesmuseum Darmstadt, Darmstadt, Germany; **SMF**, Senckenberg Naturmuseum Frankfurt, Frankfurt, Germany; **SMIF**, Shared Materials Instrumentations Facility, Duke University, Durham, North Carolina, USA.

## Systematic palaeontology

Testudines Batsch, 1788

Cryptodira Cope, 1868

Trionychia Baur, 1891

*Carettochelyidae* Gill, 1889

*Carettochelyinae* Williams, 1950

*Allaeochelys* Noulet, 1867

**Type species.** *Allaeochelys parayrei* Noulet, 1867.

*Allaeochelys meylani* sp. nov.

Fi﻿gures 2, 3, 4, 5, 6, 7, 8, 9, 10, 11, 12, 13, 14, 15, 16, 17.

**Fig. 2:**
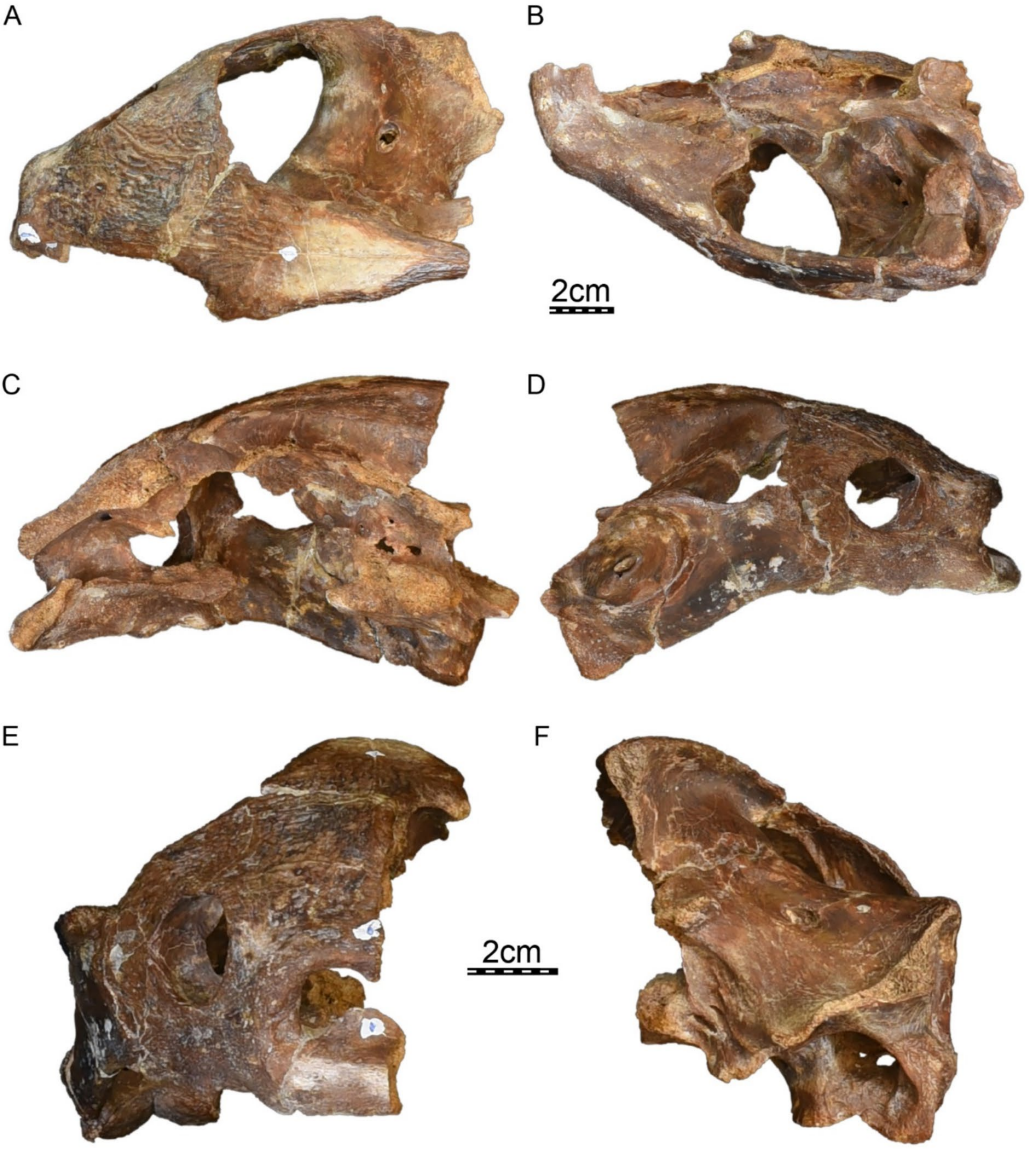
Cranium of *Allaeochelys meylani* sp. nov. (DPC 7742, holotype) from the Early Miocene (Burdigalian) Moghra Formation, Egypt. **A** dorsal view. **B** ventral view. **C** medial view. **D** right lateral view. **E** anterior view. **F** posterior view

**Holotype.** DPC 7742, a partial cranium (Fig. 2).

**Type locality.** MGL–23, Moghra, Egypt.

**Type stratum.** Lower units and F1 horizon.

**Nomenclatural acts.** This publication and its nomenclatural acts were registered at ZooBank on December 13, 2024, prior to publication. The LSID of the publication is urn:lsid:zoobank.org:pub:508B7DE2-52CF-4700-9C5D-B2526A330100 and that of the new species urn:lsid:zoobank.org:act:794EBD66-86D1-48D9-807D-CA48EE1D3960.

**Diagnosis.** *Allaeochelys meylani* sp. nov. can be diagnosed as a representative of *Carettochelyidae* by a bone surface ornamentation made up of thick ridges separated by equally sized grooves, presence of a quadrate fossa, presence of a midline carapacial keel, paired nuchal processes, and presence of a lip formed on the visceral side of the posterior peripherals and pygal. *Allaeochelys meylani* sp. nov. can furthermore be diagnosed as a member of *Carettochelyinae* by the complete loss of carapacial scutes, a contact between the maxilla and quadratojugal, a foramen posterius canalis carotici interni distant from the posterior border of the parabasisphenoid, and a deep quadrate fossa. *Allaeochelys meylani* sp. nov. can be differentiated from other carettochelyines by having exceptionally thick cranial bones, a broad bony wall posterior to the orbit, a large fossa formed by the maxilla and premaxilla at the anterior third of the triturating surface, an enlarged foramen arteriomandibulare, a shallow recess in the quadrate formed anterodorsal to the incisura columella auris, a fenestra ovalis completely surrounded by the prootic and opisthotic, a reduced hiatus acusticus, a greatly thickened parabasisphenoid with a tall posterior contact with the basioccipital, a single external foramen for the hypoglossal nerve, a subtle dorsal embayment of the common crus, notably large body size, and presence of a medial process on peripheral II.

**Referred material.** Early Miocene (Burdigalian), Moghra Formation: CGM 67151, a carapace fragment that includes a partial nuchal, partial left costal I, and left peripherals I and II; DPC 3622, a partial nuchal; DPC 6436, a left peripheral IX; DPC 7741, a pygal; DPC 12585, a partial right peripheral VI; DPC 12637, a left peripheral IV; DPC 14555, costals VIII and suprapygal; CGM 67140, a plastron fragment that includes a partial left hypoplastron and a partial left xiphiplastron. Early Miocene (Burdigalian), Wadi Faregh: BSPG unnumbered, a left peripheral I and partial nuchal (Dacqué, 1912), now lost.

**Etymology.** The eponym ‘meylani’ is created in honor of Peter A. Meylan, an eminent turtle paleontologist who provided foundational studies on trionychian anatomy, systematics, and phylogeny (Meylan, 1987, 1988).

### Description

#### Cranium

**General comments.** The cranium of DPC 7742 (Fig. 2) lacks its left half with the exception of the dorsomedial aspect of the left parietal, a small portion of the left frontal, and a tiny fragment that belongs to the left prefrontal. While the right dorsal skull roof is relatively complete, many structures are missing from the right ventral side and the midline, including the palatines, the complete vomer and parabasisphenoid, much of the pterygoid, basioccipital, and supraoccipital, and the complete squamosal.

**Fig. 3:**
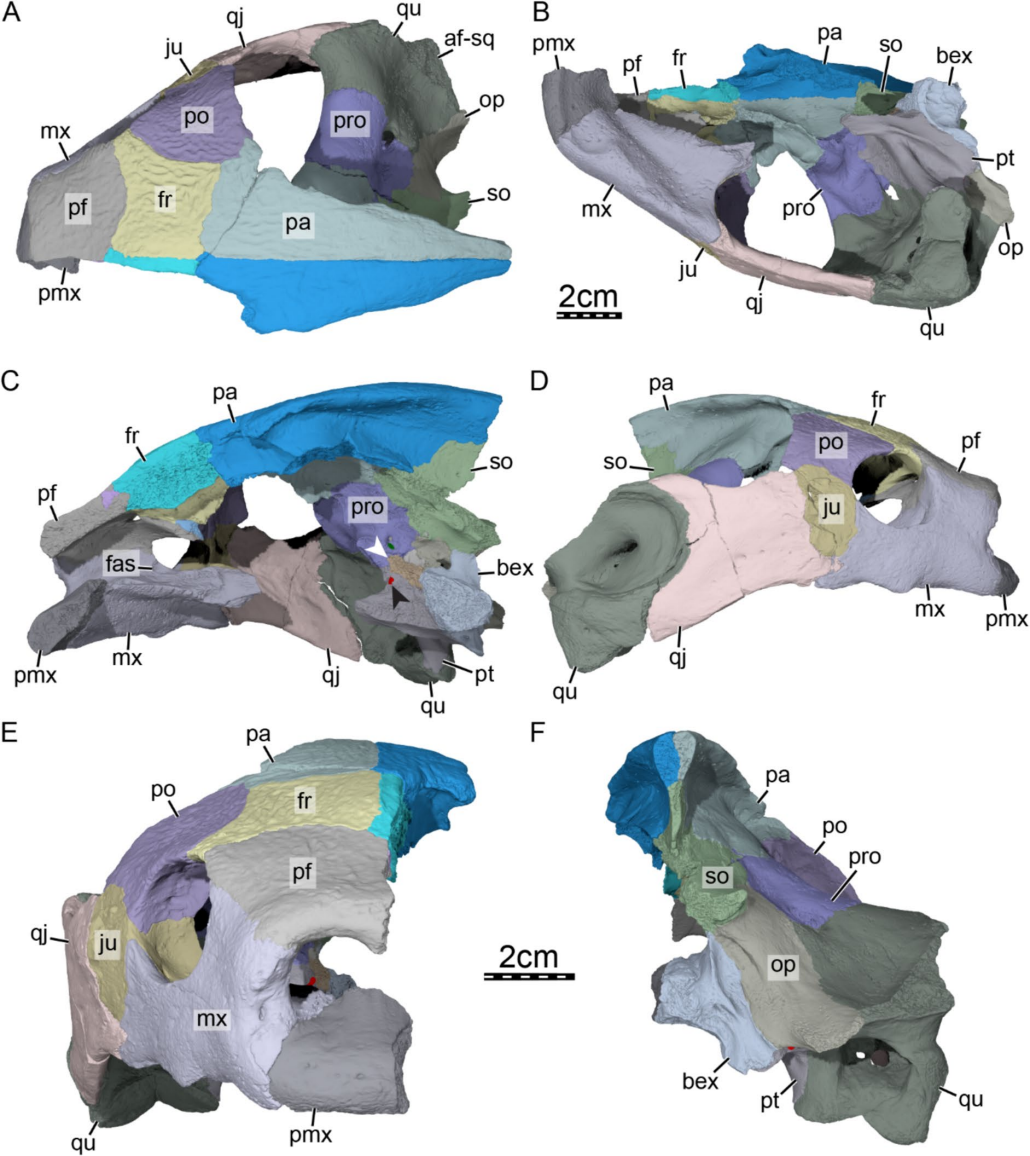
Three dimensional renderings of the cranium of *Allaeochelys meylani* sp. nov. (DPC 7742, holotype) from the Early Miocene (Burdigalian) Moghra Formation, Egypt. **A** dorsal view. **B** ventral view. **C** medial view. **D** right lateral view. **E** anterior view. **F** posterior view. *af-sq* articular facet for squamosal, *bex* basioccipital-exoccipital complex, *fas* foramen alveolare superius, *fr* frontal, *ju* jugal, *mx* maxilla, *op* opisthotic, *pa* parietal, *pf* prefrontal, *pmx* premaxilla, *po* postorbital, *pro* prootic, *pt* pterygoid, *qj* quadratojugal, *qu* quadrate, *so* supraoccipital. Black arrowhead in C indicates the canalis caroticus internus. White arrowhead in C indicates the parabasisphenoid

The skull roof of DPC 7742 exhibits the typical ornamentation of carettochelyids consisting of thick ridges separated by equally sized grooves (Fig. 2A; Joyce, 2014; Skutschas et al., 2017). The specimen presents several additional features observed in other carettochelyids, such as a deep upper temporal emargination, a posteriorly enclosed incisura columella auris, a contact between the maxilla and quadratojugal, a low mandibular condyle, and the presence of a fossa that posteriorly excavates the quadrate (Fig. 2; Danilov et al., 2017; Harrassowitz, 1922; Havlik et al., 2014; Joyce et al., 2018; Waite, 1905; Walther, 1922; White et al., 2023). DPC 7742 is larger than any other previously described carettochelyid cranium (Danilov et al., 2017; Harrassowitz, 1922; Havlik et al., 2014; Joyce et al., 2018; Waite, 1905; Walther, 1922; White et al., 2023), with an approximate length of 15 cm (cm) from the anterior margin of the prefrontals to the most posterior preserved aspect of the parietals. By comparison to *Carettochelys insculpta* (NHMUK 1903.7.10.1), a total length of about 19.5 cm is estimated from the anterior margin of the prefrontals to posterior tip of the crista supraoccipitalis, suggesting a shell length of about 68 cm by reference to the same *C. insculpta* specimen. The cranium of DPC 7742 is less gracile than that of *Anosteira pulchra* (Joyce et al., 2018) and overall similar in shape to that of *Anosteira maomingensis* (Danilov et al., 2017), *Allaeochelys libyca* (Havlik et al., 2014), *Carettochelys niahensis* (White et al., 2023), and *Carettochelys insculpta* (Joyce, 2014; Rollot et al., 2024a; Waite, 1905). The specimen is notable in comparison to all previously described specimens by exhibiting exceptionally thick bones (Fig. 2C, E). Despite damage to the cranium, the posterolateral and posteromedial portions of the basioccipital-exoccipitals complex and pterygoid, respectively, form a broad base that suggests the former presence of elongate tubercula basioccipitale, much as in other carettochelyids. The small size of the orbits relative to that of the cranium, the fusion between the basioccipital and exoccipitals, the general size of the cranium (approximately 20 cm when complete), and the thickness of bones suggest that DPC 7742 belongs to a skeletally mature individual (Figs. 2 & 3).

**Nasal.** The nasals are absent in DPC 7742, as in other carettochelyids (Fig. 2A; Danilov et al., 2017; Harrassowitz, 1922; Havlik et al., 2014; Joyce et al., 2018; Waite, 1905; Walther, 1922; White et al., 2023).

**Fig. 4:**
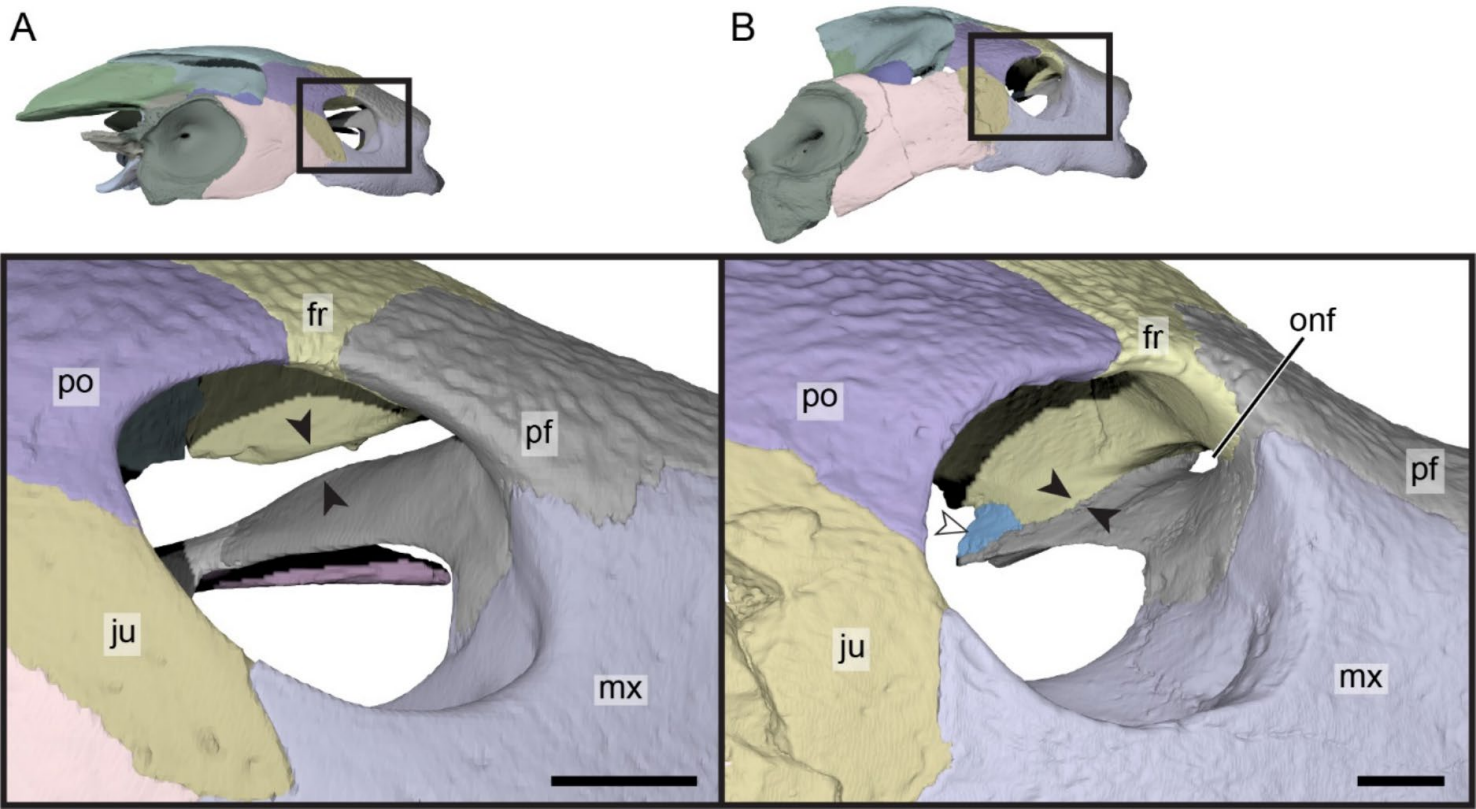
Three-dimensional renderings of the right orbital area of the extant *Carettochelys insculpta* (NHMUK 1903.7.10.1) and *Allaeochelys meylani* sp. nov. (DPC 7742, holotype) from the Early Miocene (Burdigalian) Moghra Formation, Egypt. **A** right orbital area of *Carettochelys insculpta* in lateral view, showing the absence of contact between the crista cranii of the frontal and descending process of the prefrontal. **B** right orbital area of *Allaeochelys meylani* sp. nov. in lateral view, showing the elongate contact between the crista cranii of the frontal and descending process of the prefrontal. *fr* frontal, *ju* jugal, mx maxilla, *onf* ophthalmic nerve foramen, *pf* prefrontal, *po* postorbital. White arrowhead shows the additional bone. Scale bars: 5 mm

**Prefrontal.** The prefrontal forms the anterodorsal aspect of the cranium (Fig. 3A–D). The notably thick dorsal plate of the prefrontal defines the dorsal margin of the external nares, roofs the fossa nasalis, and forms a minor contribution to the dorsal margin of the orbit (Fig. 3A, D, E). The dorsal plate broadly contacts the maxilla ventrolaterally along a straight horizontal suture, the frontal posteriorly along a slightly convex suture, and its counterpart medially (Fig. 3A & E). The prefrontal, maxilla, and premaxilla jointly form the unusually thickened margins of the external nares (Fig. 3E). Thickened anterior margins contrast with the morphology observed in other carettochelyids, where the margins of the external nares are thin or barely thickened (Danilov et al., 2017; Joyce et al., 2018; Rollot et al., 2024a, 2024b; Waite, 1905; Walther, 1922; White et al., 2023). Although the contribution of the prefrontal to the orbit appears to be relatively large in dorsal view, its contribution to the margin of the orbit per se is extremely small. This is mostly caused by a constriction of the prefrontal exposure along the orbit margin formed by the frontal posteriorly and the maxilla anteriorly, the two latter bones approaching one another very closely in this region (Fig. 3D). The descending process of the prefrontal of DPC 7742 laterally contacts the ascending process of the maxilla within the orbit along a broad and long suture that extends from the anterior limit of the foramen orbito-nasale to the anterior margin of the orbit (Fig. 3D, E). The process contributes to the anterodorsal and dorsal margin of the large foramen orbito-nasale. Posteriorly, the descending process of the prefrontal forms a protruding sheet of bone that is posteriorly damaged (Fig. 3D). This sheet likely had a posterior contact with the palatine and vomer, as in *Carettochelys insculpta* (Rollot et al., 2024a). The dorsal surface of this posterior bony process contacts the crista cranii, thereby delimiting an oval secondary foramen between the fossa orbitalis and fossa nasalis. In *Carettochelys insculpta*, such a foramen is not present, but the crista cranii of the frontal closely approaches the descending process of the prefrontal, forming a slit-like passage between the orbital and nasal fossae (Fig. 4). This slit was recently named the ophthalmic slit because it serves as the passage for the ophthalmic branch of the trigeminal nerve (CN V_1_; Rollot et al., 2024a). As the foramen observed in the anterodorsomedial corner of the orbit of DPC 7742 very likely had the same purpose as the ophthalmic slit in *Carettochelys insculpta*, we propose to name the foramen in DPC 7742 “ophthalmic nerve foramen.” An ophthalmic nerve foramen is also present in *Allaeochelys libyca* (Rollot et al., 2024b) and *Carettochelys niahensis* (White et al., 2023), but likely not in *Anosteira pulchra* (Joyce et al., 2018), in which a slit-like passage seems to be preserved. Along the posterodorsal aspect of the posterior extension of the descending process, the prefrontal contacts a small piece of bone which inserts between it and the crista cranii of the frontal (Fig. 4B). A supplementary bone can occasionally be identified between the prefrontal and vomer in *Carettochelys insculpta*. In NHMUK 1903.7.10.1 it is present only on the left side and occupies a space that on the other side is taken by the most posterior aspect of the descending process of the prefrontal. In DPC 7742, the additional bone is located slightly more dorsal and appears to be blockier in shape, but is found in a similar position to that of NHMUK 1903.7.0.1. We thus speculate that this small bony unit represents an ossification anomaly that might be related to the prefrontal. It is unlikely that this additional bone belongs to the palatine or the vomer, as the former bone lacks such an anterior extension in other carettochelyids, and the latter bears low dorsolateral processes that do not extend dorsally to such a degree (Fig. 4B).

**Frontal.** The frontal is a nearly square bone that forms the dorsomedial portion of the skull roof (Fig. 3A). The thickened dorsal plate of the frontal contacts the prefrontal anteriorly along a concave suture, the postorbital laterally along a concave suture, the dorsal plate of the parietal posteriorly along a straight transversal suture, and its counterpart medially (Fig. 3A &D–E). The frontal forms a short process anterolaterally that contributes slightly to the orbit margin and prevents the prefrontal and postorbital from contacting one another (Fig. 3A & D–E). Ventrally, the frontal forms a robust, dorsoventrally expanded crista cranii (Figs. 3D & 4B). The crista cranii forms the lateral wall of the rounded sulcus olfactorius, which is tall and relatively short, and appears to be slightly narrower than that of *Allaeochelys libyca* (Rollot et al., 2024b), *Carettochelys niahensis* (White et al., 2023), and *Carettochelys insculpta* (Rollot et al., 2024a). Within the roof of the orbit, the crista cranii laterally forms a series of small foramina with unknown affinities. A low ridge extends anterodorsally from the lateral surface of the crista cranii and parasagittally along the most anterior aspect of the frontal ventral surface, which delineates a depression in the roof of the orbit, as in *Carettochelys insculpta*, albeit deeper (Rollot et al., 2024a). Anteroventrally, the crista cranii contacts the posterior aspect of the descending process of the prefrontal, and collectively with the latter forms the ophthalmic nerve foramen (Figs. 3D & 4B), as in *Allaeochelys libyca* (Rollot et al., 2024b) and *Carettochelys niahensis* (White et al., 2023). This contact between the crista cranii and descending process of the prefrontal is anteroposteriorly elongate in DPC 7742 and appears to be greater than that observed in *Allaeochelys libyca* (Rollot et al., 2024b) but is overall similar to that of *Carettochelys niahensis* (White et al., 2023). The posteroventral part of the crista cranii is continuous with the descending process of the parietal and both bones extend relatively far ventrally, so that the foramen interorbitale was likely greatly reduced in size (Fig. 3C). A similar arrangement was likely present in *Carettochelys niahensis* (White et al., 2023), but not in *Anosteira pulchra* (Joyce et al., 2018) and *Carettochelys insculpta* (Rollot et al., 2024a; Waite, 1905; Walther, 1922).

**Parietal.** The left parietal is heavily damaged, but its right counterpart preserves most of its anatomy, with the exception of the most posterior and anteroventrolateral portions (Fig. 3A–C). On the skull roof, the thickened parietal contacts the frontal anteriorly along a transverse suture, the postorbital anterolaterally, and its counterpart medially (Fig. 3A). Within the upper temporal fossa, the parietal contacts the prootic ventrolaterally and the supraoccipital posteroventrolaterally (Fig. 3A, D & F). The parietal forms the medial and anteromedial margins of the upper temporal emargination, which is deep as in other carettochelyids (Danilov et al., 2017; Havlik et al., 2014; Joyce et al., 2018; Waite, 1905; Walther, 1922; White et al., 2023). The ventral aspects of the descending process of the parietal, or processus inferior parietalis, are damaged on both sides of the specimen. The remaining, dorsal aspects of the descending process, however, are developed as a ventrally expanded sheet of bone that forms the anterolateral part of the secondary braincase wall and is anteriorly continuous with the crista cranii of the frontal. The parietal forms the medial portion of the well-developed processus trochlearis oticum, which constitutes a distinct overhanging lip best seen in ventral view (Fig. 3A–B). This lip extends anteriorly along the ventrolateral and lateral surface of the postorbital and jugal, respectively, as in *Carettochelys insculpta*, but less distinctly so (Rollot et al., 2024a). Ventromedial to this lip and continuous with the processus trochlearis oticum, two shallow fossae can be identified along the lateral surface of the braincase wall (Fig. 3B). The anterior fossa is formed by the parietal, located just posterior to the suture with the frontal, and is anterolaterally bordered by a shallow ridge formed by the parietal and frontal. The posterior fossa is encompassed by the prootic and parietal and is larger than the anterior one. Such fossae were not reported for other carettochelyids, but their shallow nature might complicate their recognition in many fossils. Nevertheless, a similar anterior fossa is present in *Allaeochelys libyca* (Rollot et al., 2024b), but both fossae are clearly absent in *Carettochelys insculpta* (Rollot et al., 2024a). The braincase is greatly enlarged anterolaterally suggesting the development of broad cerebral hemispheres (Fig. 3B), as in *Carettochelys insculpta* (Rollot et al., 2024a). The most anterior section of the braincase, i.e. the area that housed the olfactory bulbs, is notably constricted in DPC 7742, and narrower than in *Allaeochelys libyca* (Rollot et al., 2024b) and *Carettochelys insculpta* (Rollot et al., 2024a). At the level of its contact with the supraoccipital within the braincase, a dorsoventral constriction of the braincase is apparent, corresponding to the cartilaginous rider (Werneburg et al., 2021). Additional contacts of the parietal with the pterygoid and epipterygoid are generally present in carettochelyids, but the damage that affects the anteroventral portions of the descending process of the parietal prevents us from making any further statements.

**Postorbital.** The postorbital forms the posterior margin of the orbit and the anterior margin of the deep upper temporal emargination (Fig. 3A & D–E). The postorbital contacts the frontal laterally along a rounded, convex suture, the jugal ventrolaterally, the quadratojugal posteroventrolaterally, and the parietal posteromedially (Fig. 3A & D–E). The ventrolateral half of the postorbital is notably enlarged (Fig. 3E), forming the dorsal part of a thin but mediolaterally expanded bony wall posterior to the orbit that is reminiscent of the septum orbitotemporale seen in pleurodires (sensu Gaffney et al., 2006) and trionychids. This septum is buttressed by a ventral ridge that is continuous with the ventral ridges of the parietal medially and the jugal ventrally, as in *Carettochelys insculpta* (Rollot et al., 2024a). At about one third of its length, the ridge that crosses the ventral surface of the postorbital gives off a short accessory ridge that extends anterodorsally (Fig. 3B). Those two ridges collectively divide the ventral surface of the postorbital into three distinct, concave areas: one facing anteroventrally, one facing anteromedially, and one facing posteriorly.

**Jugal.** The jugal is a relatively small bone that forms the posteroventral wall of the orbit and the anterior margin of the lower temporal fossa collectively with the maxilla (Fig. 3D–E). On the external surface, the jugal contacts the postorbital dorsally, the maxilla anteroventrally, and the quadratojugal posteriorly and posteroventrally (Fig. 3D–E). Medially, an additional contact between the jugal and palatine, as seen in *Carettochelys insculpta*, might have occurred, but this cannot be ascertained with certainty as the palatine is missing. The contribution of the jugal to the orbit margin is greatly restricted by a descending process of the postorbital and an ascending process of the maxilla (Fig. 3D–E). This contrasts with available specimens of *Anosteira maomingensis* (Danilov et al., 2017), *Anosteira pulchra* (Joyce et al., 2018), and *Carettochelys niahensis* (White et al., 2023), where the jugal forms most of the posterior or posteroventral margin of the orbit. In *Carettochelys insculpta*, the degree of contribution of the jugal to the orbit margin appears to be quite variable as affected by both ontogenetic and individual variation, with larger individuals having a reduced contribution (Rollot et al., 2024a; Walther, 1922). The jugal of DPC 7742 forms a well-developed medial process that broadly overlays the maxilla and forms the ventral half of the septum orbitotemporale, which is otherwise formed by the postorbital (Fig. 3E).

**Quadratojugal.** The quadratojugal is a large, plate-like bone that forms the lateral margin of the deep upper temporal emargination (Fig. 3A & D). The quadratojugal contacts the quadrate posteriorly along a deeply concave suture, the postorbital anterodorsally, the jugal anteriorly, and the maxilla anteroventrally below the jugal (Fig. 3A & D–E), as in *Carettochelys niahensis* (White et al., 2023) and *Carettochelys insculpta* (Waite, 1905; Walther, 1922). A cheek emargination is completely absent in DPC 7742 (Fig. 3D). Although the cheek emargination is generally reduced in carettochelyids, inter- and intraspecific variation occurs and emargination varies from a moderate state in *Anosteira maomingensis* (Danilov et al., 2017) to very shallow in *Anosteira pulchra* (Joyce et al., 2018) and *Carettochelys niahensis* (White et al., 2023). In *Carettochelys insculpta*, individual variation must also be considered as cheek emargination ranges from a shallow notch (see Rollot et al., 2024a) to complete absence (Waite, 1905). The quadratojugal in DPC 7742 does not contribute to the cavum tympani (Fig. 3D). The posterodorsal process of the quadratojugal is short and barely extends above the cavum tympani (Fig. 3D). A posterior contact with the squamosal was likely absent, as in other carettochelyids, as the dorsal surface of the quadrate just posterior to the posterodorsal process of the quadratojugal is smooth and lacks an articular facet. The posteroventral process of the quadratojugal is short and posteriorly ends slightly anterior to the level of the incisura columella auris (Fig. 3D). The process is dorsoventrally tall, much as in *Allaeochelys libyca* (Havlik et al., 2014; Rollot et al., 2024b), but unlike *Carettochelys insculpta*, where it ends in a low, pointed process (Rollot et al., 2024a).

**Squamosal.** The squamosals are not preserved in DPC 7742.

**Premaxilla.** Although the premaxilla is damaged in DPC 7742, it is complete enough to allow confirmation that it is a single, fused, midline element that is missing most of its left half (Fig. 3E). The premaxilla is a small but thick bone that forms the ventral margin of the external nares and floors the fossa nasalis anteriorly (Fig. 3E). The only preserved contact of the premaxilla is lateral with the maxilla (Fig. 3E). We are not able to assess other possible contacts of the premaxilla as its medial and posterior aspects are missing, but comparison with *Carettochelys insculpta* suggests that none were present. Together with the maxilla and the prefrontal, the premaxilla forms the greatly thickened margins of the external nares (Fig. 3E). This thickening is unique among carettochelyids, as the margin of the external nares is otherwise either thin or only slightly thickened (Danilov et al., 2017; Joyce et al., 2018; Rollot et al., 2024a, 2024b; Waite, 1905; Walther, 1922; White et al., 2023). The foramen praepalatinum is not developed, as in other carettochelyids (Danilov et al., 2017; Rollot et al., 2024a; Waite, 1905; Walther, 1922). Instead, the premaxilla anteriorly frames the enlarged foramen intermaxillaris (Fig. 3B).

**Fig. 5:**
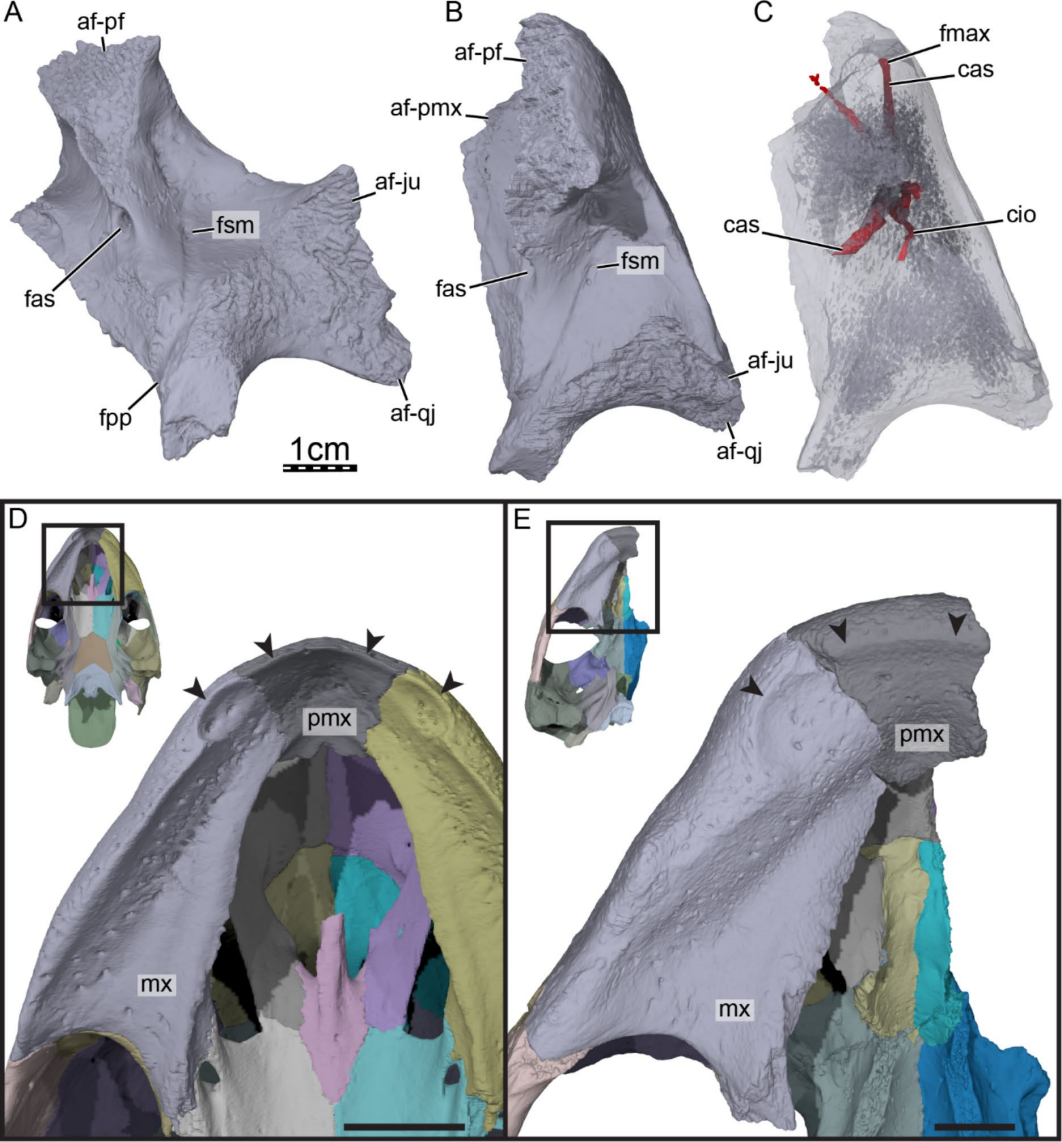
Three-dimensional renderings of digitally reconstructed bones from the anterior portion of the cranium of the extant *Carettochelys insculpta* (NHMUK 1903.7.10.1) and *Allaeochelys meylani* sp. nov. (DPC 7742, holotype) from the Early Miocene (Burdigalian) Moghra Formation, Egypt. **A** posteromedial view of the right maxilla of *Allaeochelys meylani* sp. nov. **B** dorsal view of the right maxilla of *Allaeochelys meylani* sp. nov. **C** dorsal view of the right maxilla of *Allaeochelys meylani* sp. nov. showing the path of arterial canals. **D** triturating surface of *Carettochelys insculpta*. **E** triturating surface of *Allaeochelys meylani* sp. nov. *af-ju* articular facet for jugal, *af-pf* articular facet for prefrontal, *af-pmx* articular facet for premaxilla, *af-qj* articular facet for quadratojugal, *cas* canalis alveolaris superior, *cio* canalis infraorbitalis, *fas* foramen alveolare superius, *fmax* maxillary foramen, *fpp* foramen palatinum posterius, *fsm* foramen supramaxillare, *mx* maxilla, *pmx* premaxilla. Black arrowheads indicate the half-cup-like fossa along the anterior part of the triturating surface. Scale bars: 1 cm

**Maxilla.** The right maxilla is preserved almost fully intact. On the external surface, the maxilla contacts the premaxilla anteromedially, the prefrontal dorsally, the jugal posteriorly, and the quadratojugal posteroventrally (Fig. 3D–E). Articular scars and a comparison with *Carettochelys insculpta* suggest the maxilla may have had additional contacts with the palatine and pterygoid. Anteriorly, the maxilla forms the lateral margin of the external nares, which are extremely thickened (Fig. 3E). This morphology is unique among carettochelyids, as this area is either thin or barely thickened in other taxa (Danilov et al., 2017; Joyce et al., 2018; Rollot et al., 2024a, 2024b; Waite, 1905; Walther, 1922; White et al., 2023). The maxilla forms the anterior and ventral margin of the orbit and the anteroventral and ventral margin of the large foramen orbito-nasale (Fig. 3D). The maxilla floors much of the fossa orbitalis and forms the foramen supramaxillare in the anterior third of the fossa (Fig. 5A, B; sensu Albrecht, 1967). The foramen supramaxillare leads into the canalis infraorbitalis, which extends anteroventrolaterally through the maxilla (Fig. 5C). At about the same level as the foramen supramaxillare, the maxilla forms the foramen alveolare superius, which is located within the nasal cavity just anteroventromedial to the foramen orbito-nasale (Fig. 5A, B), as in *Carettochelys insculpta*. The foramen alveolare superius leads into the canalis alveolaris superior, which extends anterolaterally and merges with the canalis infraorbitalis (Fig. 5C). The resulting canal retains the name canalis alveolaris superior (sensu Albrecht, 1967) and extends anteriorly through the maxilla. Several canals branch from the canalis alveolaris superior to its course towards the most anterior portion of the cranium, and we here report the major accessory canals that were identified in the CT scans. Just anterior to the point where the canalis alveolaris superior merges with the canalis infraorbitalis, two canals successively branch off the canalis alveolaris superior at the level of the anterior margin of the orbit. These canals extend dorsally within the maxilla and exit the cranium along the anterior wall of the fossa orbitalis (Fig. 5B, C). At about mid-length between the external nares and anterior margin of the orbit, another canal branches off the canalis alveolaris superior and extends anteromedially through the maxilla and premaxilla, but its anterior exit remains unclear in the CT scans. In *Carettochelys insculpta*, however, this canal exits along the ventral margin of the external nares at the suture between the maxilla and premaxilla (Rollot et al., 2024a). Anteriorly, the canalis alveolaris superior of DPC 7742 exits the skull by means of a maxillary foramen located along the ventrolateral margin of the external nares (Figs. 3E & 5C). This foramen is relatively small and is also present in *Carettochelys insculpta* (Rollot et al., 2024a) and likely *Carettochelys niahensis* (White et al., 2023). The abovementioned two canals and their respective foramina very likely contained branches of the superior alveolar artery, as in *Carettochelys insculpta.* The superior alveolar artery enters the foramen alveolare superius and extends anteriorly through the canalis alveolaris superior (Rollot et al., 2024a). The position of an anterior foramen for the canalis alveolaris superior along the lateral margin of the external nares seen in carettochelyids is quite unusual for turtles, as the canalis alveolaris superior generally gives off numerous, small accessory canals that connect to the ventrolateral surface of the maxilla and the triturating surface, and anteriorly ends within the maxilla and premaxilla in a series of very small canals that either become mixed up with the porosity of the bones or join the anterior and ventral surfaces of these bones. *Carettochelys insculpta* possesses a protruding, fleshy snout (Walther, 1922), and the location of a foramen for the passage of a branch of the superior alveolar artery along the ventrolateral margin of the bony external nares may be linked to the need to supply the proboscis with blood, relative to other turtles that lack a proboscis. Posterior to the foramen supramaxillare, the maxilla forms a groove in the floor of the fossa orbitalis that extends posteromedially from the foramen, closely approaching the maxilla-jugal suture in the posteroventromedial corner of the orbit, and extending towards ventral along the posteromedial surface of the maxilla (Fig. 5A). This vertical portion of the groove corresponds to the location of the foramen palatinum posterius in other carettochelyids (Danilov et al., 2017; Joyce et al., 2018; Waite, 1905; Walther, 1922), and we therefore interpret this passage as such in DPC 7742. Given the inferred position of the foramen palatinum posterius, the foramen was likely formed by the maxilla and palatine as in *Anosteira maomingensis* (Danilov et al., 2017) and *Anosteira pulchra* (Joyce et al., 2018). In *Carettochelys insculpta*, however, bony contributions to the foramen palatinum posterius are variable and subject to ontogenetic variation as the foramen is formed by the palatine and maxilla in juveniles but only by the palatine in adults (Rollot et al., 2024a). Individual variation might also affect which bones are contributing to the foramen palatinum posterius, as the pterygoid closely approaches the foramen in other specimens, but this remains unclear as detailed descriptions are lacking for the specimens of interest (see Waite, 1905; Walther, 1922).

The triturating surfaces of DPC 7742 are highly unusual, not only for carettochelyids specifically, but also among turtles in general. In lateral view, the ventral margin of the maxilla forms a wavy labial margin, which is partially created by the development of a low, tooth-like cusp below the eye (Fig. 3D). In ventral view, the low but broad labial ridge jointly formed by the premaxilla and maxilla is not straight for its entire length, as in carettochelyids. Instead, the labial ridge forms a short process at about one-third of its anteroposterior length that protrudes medially into the triturating surface and separates it into a half-cup-like fossa anteriorly and a triangular groove posteriorly (Fig. 5D, E). The fossa is formed by the most anterior part of the maxilla and the whole premaxilla. A nuanced fossa is present in *Carettochelys insculpta* (Rollot et al., 2024a), but not in *Anosteira maomingensis* (Danilov et al., 2017) and *Anosteira pulchra* (Joyce et al., 2018). A closer look at the CT scans of the recently published stained juvenile specimen of *Carettochelys insculpta* USNM 327960 (see MorphoSource; Rollot et al., 2024a) reveals that the shape of this fossa is reflected in the shape of the rhamphotheca. In USNM 327960, the ventral, cutting edge of the rhamphotheca is not straight but rather forms a slight lateral excavation for a short distance at the level of the posterior part of the fossa formed by the maxilla. We therefore suggest that a similar structure was present on the rhamphotheca in DPC 7742. As the fossa is larger than that identified in *Carettochelys insculpta* (adult or juvenile; Rollot et al., 2024a), it is very likely that the rhamphotheca of DPC 7742 was excavated to a greater degree than in *Carettochelys insculpta*. Although little is known about the anatomy of the rhamphotheca in turtles, we hypothesize that the function of this excavation in the rhamphotheca was to allow the processing of specific types of food. *Carettochelys insculpta* is a generalist omnivore with a predilection towards herbivory (Georges & Kennett, 1989; Heaphy, 1990), and the similarity between the labial ridges and triturating surfaces of *Carettochelys insculpta* and DPC 7742 highlight that the two taxa likely had similar diets. The presence of a hollow on the rhamphotheca might represent a functional advantage in shearing fibrous food, but further studies on the anatomy of turtle rhamphothecae are needed to test this hypothesis.

**Palatine.** The palatines are not preserved in DPC 7742.

**Vomer.** The vomer is not preserved in DPC 7742.

**Fig. 6:**
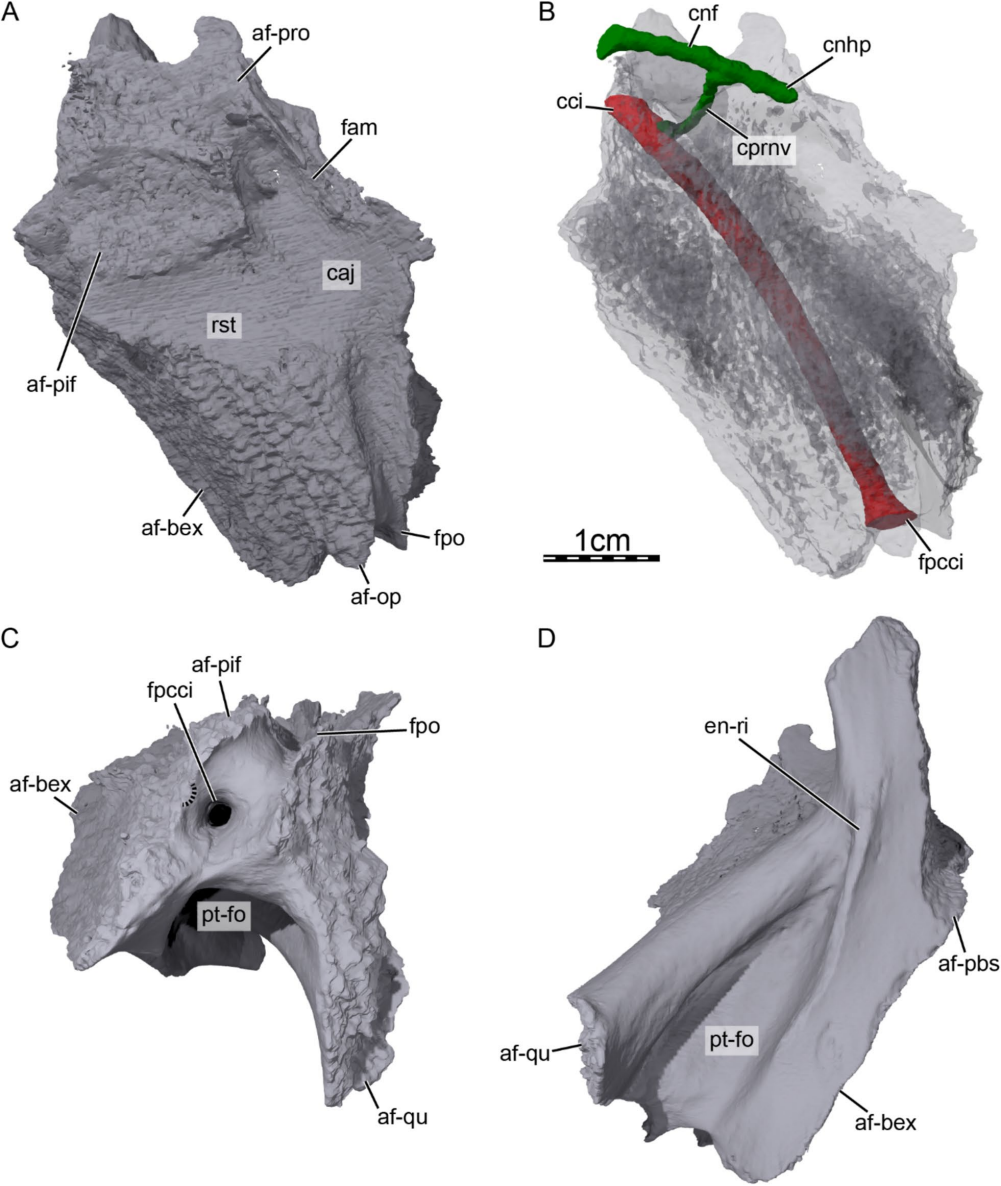
Three-dimensional renderings of the right pterygoid of *Allaeochelys meylani* sp. nov. (DPC 7742, holotype) from the Early Miocene (Burdigalian) Moghra Formation, Egypt. **A** dorsal view. **B** dorsal view rendered transparent to show the circulatory and innervation systems. **C** posterior view. **D** ventral view. *af-bex* articular facet for basioccipital-exoccipital complex, *af-op* articular facet for opisthotic, *af-pbs* articular facet for parabasisphenoid, *af-pif* articular facet for processus interfenestralis of the opisthotic, *af-pro* articular facet for prootic, *af-qu* articular facet for quadrate, *caj* cavum acustico-jugulare, *cci* canalis caroticus internus, *cnf* canalis nervus facialis, *cnhp* canalis nervus hyomandibularis proximalis, *cprnv* canalis pro ramo nervi vidiani, *en-ri* enfolded ridge, *fam* foramen arteriomandibulare, *fpcci* foramen posterius canalis carotici interni, *fpo* fenestra postotica, *pt-fo* pterygoid fossa, *rst* recessus scalae tympani. Dashed lines in C indicate the lateral margin of the foramen oropharyngeale

**Pterygoid.** Only the posterior half of the right pterygoid is preserved in DPC 7742 (Figs. 3B & 6). The preserved portion of the pterygoid contacts the basioccipital-exoccipital complex posteromedially, the quadrate posterolaterally and laterally, the opisthotic posterodorsally and dorsally, and the prootic anterodorsally (Fig. 3B–C & F). The medial surface of the pterygoid and anteroventral surface of the basioccipital-exoccipital complex are intact and show the medial articulation facets with the parabasisphenoid (Fig. 6D). The ventral surface of the posterior half of the pterygoid is excavated to form a deep pterygoid fossa (Fig. 6C, D), as in *Anosteira maomingensis* (Danilov et al., 2017), *Allaeochelys crassesculpta* (Harrassowitz, 1922), *Allaeochelys libyca* (Havlik et al., 2014; Rollot et al., 2024b), and *Carettochelys insculpta* (Waite, 1905; Walther, 1922), but not *Anosteira pulchra* (Joyce et al., 2018). The pterygoid fossa of DPC 7742 is also notably broad, as in *Anosteira maomingensis* (Danilov et al., 2017) and *Allaeochelys libyca* (Havlik et al., 2014; Rollot et al., 2024b), but not in *Carettochelys insculpta* (Rollot et al., 2024a; Waite, 1905; Walter, 1922) where the fossa is narrow. The ventromedial margin of the pterygoid fossa is marked by a low ridge that extends anteroposteriorly for about half of the preserved length of the pterygoid (Fig. 6D). The ridge is broken, but likely formed a well-developed, enfolded ridge that partially covered the pterygoid fossa ventrally, as in *Allaeochelys libyca* (Rollot et al., 2024b) and *Carettochelys insculpta* (Joyce, 2014; Walter, 1922), but not *Anosteira maomingensis* (Danilov et al., 2017) and *Anosteira pulchra* (Joyce et al., 2018), where the ridge appears to be absent. Posteriorly, the pterygoid forms the entire foramen posterius canalis carotici interni, which is located just ventrolateral to the small fenestra postotica, to which the pterygoid only contributes ventrally (Fig. 6C). The foramen posterius canalis carotici interni is hidden from ventral view (Fig. 6D), as in *Allaeochelys libyca* (Rollot et al., 2024b). The foramen posterius canalis carotici interni leads into the canalis caroticus internus, which is a long canal oriented anteromedially that crosses the full preserved portion of the pterygoid (Fig. 6B). The anterior third of the canalis caroticus internus is more medially inclined than the rest of the canal, and the anterior end of the preserved portion of the canal is located at the level of the inferred sutural contact between the pterygoid and parabasisphenoid. The missing portion of the canal anteriorly corresponds to the canalis caroticus basisphenoidalis, and the canalis caroticus internus is completely preserved and apparently longer than that of *Allaeochelys libyca* (Rollot et al., 2024b) and *Carettochelys insculpta* (Rollot et al., 2024a), mostly because it enters the skull posteriorly. The area formed by the pterygoid and that separates the foramen posterius canalis carotici interni from the fenestra postotica forms a smooth, curved bony margin (Fig. 6C), which we interpret as corresponding to the location of the split between the internal carotid artery and stapedial artery by reference to the circulatory system of *Carettochelys insculpta* (Rollot et al., 2024a). Dorsomedial to the foramen posterius canalis carotici interni, the pterygoid forms the lateral margin of a small foramen, which is medially bordered by the opisthotic (Fig. 6C). The foramen is located in the same position as the foramen oropharyngeale (sensu Evers & Al Iawati, 2024), which in *Carettochelys insculpta* (Rollot et al., 2024a) and *Allaeochelys libyca* (Rollot et al., 2024b) serves as the passage of the glossopharyngeal nerve (CN IX) from the recessus scalae tympani to the back of the skull. Here, the foramen leads into a small canal that can only be traced in the CT scans for a short distance along the pterygoid-opisthotic suture. It is likely that the canal extends further anteriorly to join the recessus scalae tympani, as in *Allaeochelys libyca* (Rollot et al., 2024b) and *Carettochelys insculpta* (Rollot et al., 2024a), but we are not able to observe it.

The pterygoid floors the cavum acustico-jugulare and recessus scalae tympani (Fig. 6A). The posterior third of the cavum acustico-jugulare is greatly constricted, forming a funnel-like passage towards the central part of the cavum (Fig. 6A). The posterodorsal surface of the pterygoid forms a low, but moderately broad and elongate bony platform that contacts the paroccipital process of the opisthotic dorsally (Fig. 6A), as in other carettochelyids (Danilov et al., 2017; Havlik et al., 2014; Joyce, 2014; Rollot et al., 2024a, 2024b). The area anterior to the recessus scalae tympani is slightly raised and forms a cup-like articulation facet for the contact of the pterygoid with the broad and expanded processus interfenestralis of the opisthotic (Fig. 6A). The anterior and lateral edge of this cup-like platform also form low ridges that embrace the anterior and lateral aspects of the processus interfenestralis (Fig. 6A). Anterior to this raised, cup-like platform, the pterygoid is inclined anteroventrally and forms an articulation facet for the dorsal contact with the prootic (Fig. 6A). The central part of the cavum acustico-jugulare is slightly expanded mediolaterally, but the anterior third of the cavum is constricted like its posterior third by raised ridges of the pterygoid medially and laterally (Fig. 6A). Anteriorly, the cavum acustico-jugulare is closed by a narrow wall of bone formed by the prootic dorsally and the pterygoid ventrally and is therefore not anteriorly continuous with the canalis cavernosus (Fig. 6A). Laterally, the pterygoid forms the anteroventral margin of the enlarged foramen arteriomandibulare, which served as a passage for the mandibular artery and lateral head vein from the cavum acustico-jugulare towards the lateral side of the cranium. Just lateral to the lateral raised ridge that delineates the anterior portion of the cavum acustico-jugulare, the pterygoid forms a narrow bony platform collectively with the prootic, which we interpret as an extremely constricted canalis cavernosus that extends between the foramen arteriomandibulare and foramen nervi trigemini, as in *Carettochelys insculpta* (Rollot et al., 2024a; see Prootic for further details).

**Epipterygoid.** Although the right trigeminal area is present, the right epipterygoid is not preserved in DPC 7742, but indirect evidence is available suggesting that it was likely present. Some portions of the anterolateral braincase wall formed by the prootic are preserved, including the posterior half of the foramen nervi trigemini *sensu stricto* (sensu Rollot et al., 2024b). The anterodorsal margin of the foramen nervi trigemini is formed by an anteroventral process of the prootic, as observed in *Allaeochelys libyca* (Rollot et al., 2024b) and *Carettochelys insculpta* (Rollot et al., 2024a). In the latter two taxa, this bony bump of the prootic also serves as an articulation facet with the dorsal edge of the epipterygoid. This suggests by analogy that the epipterygoid was present in DPC 7742 as well. Additional, albeit more equivocal evidence can be found in the area between the foramen arteriomandibulare and foramen nervi trigemini. The pterygoid and prootic form a narrow, bony platform that extends between these two foramina. According to our digital reconstructions, the anterior half of this platform is exclusively formed by the prootic, although the sutural contact between the pterygoid and prootic in that area remains unclear in the CT scans. The lateral surface of this narrow platform appears to be broken, but that could also correspond to an articulation facet as observed in *Allaeochelys libyca* (Rollot et al., 2024b) and *Carettochelys insculpta* (Rollot et al., 2024a), where the epipterygoid medially rests on the pterygoid in the same area. In DPC 7742, however, we note that the lateral surface of the prootic below the bony platform is smooth and does not exhibit a rough surface similar to the articulation facet of *Allaeochelys libyca* (Rollot et al., 2024b). As the bony arrangement between the prootic, pterygoid, and quadrate is overall similar in DPC 7742, *Allaeochelys libyca*, and *Carettochelys insculpta*, we hypothesize that the epipterygoid in DPC 7742 was likely similar in shape and had similar contacts than that of its two abovementioned relatives.

**Fig. 7:**
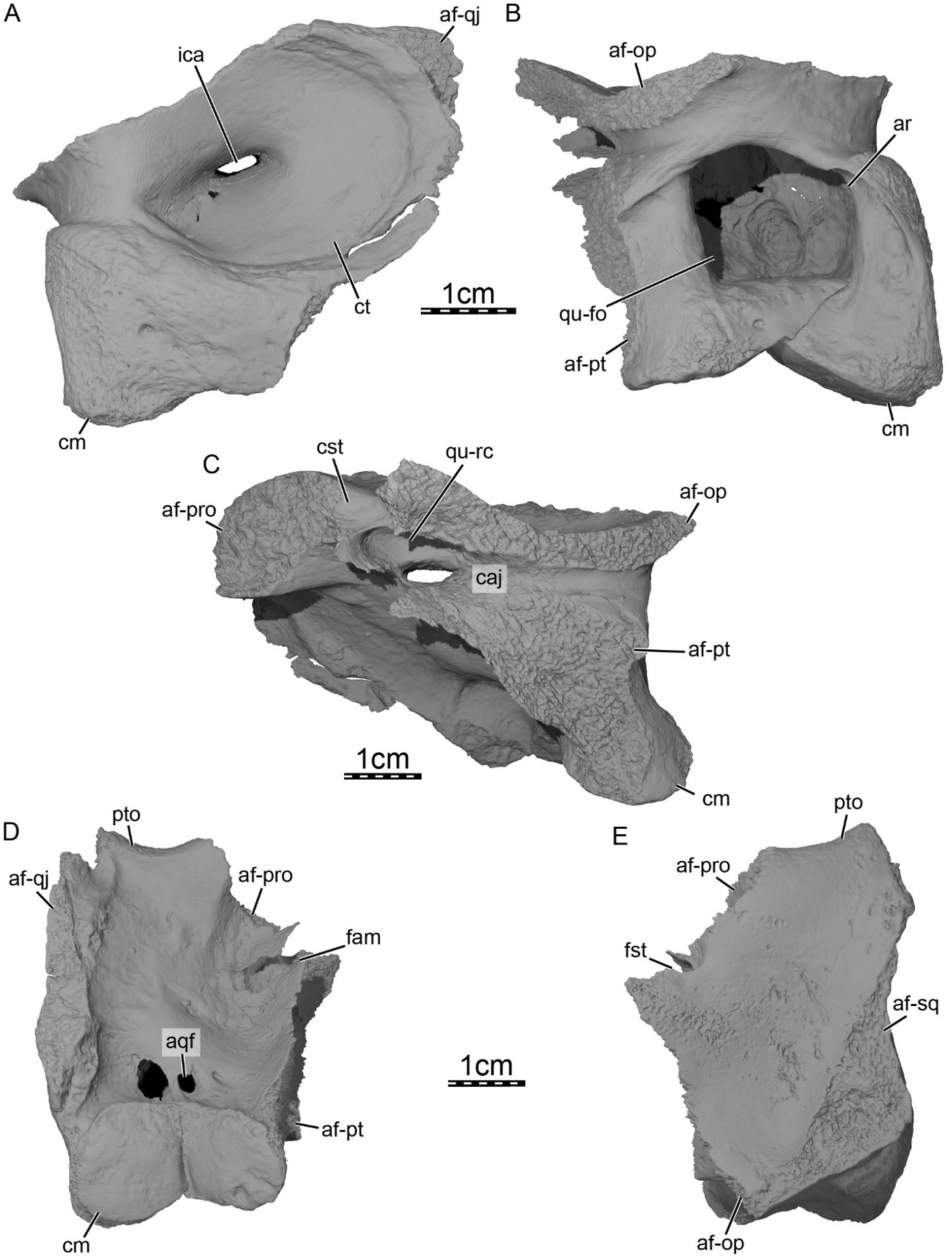
Three-dimensional renderings of the right quadrate of *Allaeochelys meylani* sp. nov. (DPC 7742, holotype) from the Early Miocene (Burdigalian) Moghra Formation, Egypt. **A** lateral view. **B** posterior view. **C** medial view. **D** ventral view. **E** dorsal view. *af-op* articular facet for opisthotic, af-pro articular facet for prootic, *af-pt* articular facet for pterygoid, *af-qj* articular facet for quadratojugal, *af-sq* articular facet for squamosal, *aqf* anterior quadrate foramen, *ar* accessory ridge, *caj* cavum acustico-jugulare, cm condylus mandibularis, *cst* canalis stapedio-temporalis, *ct* cavum tympani, *fam* foramen arteriomandibulare, *fst* foramen stapedio-temporale, ica, incisura columella auris, *pto* processus trochlearis oticum, *qu-fo* quadrate fossa, *qu-rc* quadrate recess

**Quadrate.** The quadrate forms the posterolateral aspect of the cranium, and the right element is fully preserved in DPC 7742. The quadrate forms the lateral third of the processus trochlearis oticum and the anterodorsal, anterior, and ventral margins of the cavum tympani (Fig. 7). The quadrate also fully encloses the incisura columella auris (Fig. 7A), as in other carettochelyids (Danilov et al., 2017; Havlik et al., 2014; Joyce, 2014; Joyce et al., 2018; Waite, 1905; Walther, 1922). The cavum tympani is funnel-shaped and lacks any evidence of an antrum postoticum (Fig. 7A). Though variable, at least a minor antrum postoticum is present in *Allaeochelys libyca* (Rollot et al., 2024b) and *Carettochelys insculpta* (Rollot et al., 2024a). As a result, the broad, triangular scar that articulates with the squamosal is not interrupted by the antrum postoticum in DPC 7742.

Within the upper temporal fossa, the quadrate contacts the prootic anteromedially and the opisthotic posteromedially and forms the posterolateral margin of the large foramen stapedio-temporale (Figs. 2A & 7E). Within the lower temporal fossa, the quadrate contacts the prootic anterodorsomedially, the pterygoid anteroventromedially, and the quadratojugal anterolaterally (Fig. 3B). The quadrate forms the posterior margin of the enlarged foramen arteriomandibulare, which deeply expands posteriorly within the latter bone. Although some parts of the margins formed by the quadrate are damaged (i.e. the dorsal and ventral margins), the posterior portion of the margin is smooth and appears to be intact in the CT scans, highlighting that the foramen arteriomandibulare is greatly expanded and therefore larger than that of *Allaeochelys libyca* (Rollot et al., 2024b) and *Carettochelys insculpta* (Rollot et al., 2024a). The quadrate is recessed just dorsal to the foramen arteriomandibulare, creating a fossa posteromedial to the processus trochlearis oticum, of which the anterior half is formed by the prootic (Fig. 7D). The quadrate is also slightly recessed just anterodorsal to the mandibular condyle and forms two fenestra-like passages between the lower temporal fossa and the quadrate fossa, of which the lateral passage is the largest (Fig. 7D). The medial passage occupies a similar position to the anterior quadrate foramen of *Allaeochelys libyca* (sensu Rollot et al., 2024b) and *Carettochelys insculpta* (Rollot et al., 2024a), but instead of leading into a canal that extends dorsally through the quadrate and joins the quadrate fossa, it constitutes a fenestra that directly opens into the quadrate fossa. The mandibular condyle is low and ventrally oriented as in other carettochelyids (Figs. 3D & 7A; Danilov et al., 2017; Havlik et al., 2014; Joyce et al., 2018; Waite, 1905; Walther, 1922). The lateral articular facet is larger than the medial one (Fig. 7D), also similar to other carettochelyids, although the size difference between the facets seems more significant in *Allaeochelys libyca* (Rollot et al., 2024b). A narrow and deep, centrally placed sulcus separates the two articular facets from one another, shaping the transition from one facet to the other as a steep boundary (Fig. 7B & D), which slightly differs from the more curved and smoother transition seen in *Anosteira maomingensis* (Danilov et al., 2017), *Anosteira pulchra* (Joyce et al., 2018), *Allaeochelys libyca* (Rollot et al., 2024b), and *Carettochelys insculpta* (Rollot et al., 2024a). The posterior surface of the quadrate is greatly excavated (Fig. 7B) and forms a deep quadrate fossa as in *Allaeochelys crassesculpta* (Harrassowitz, 1922) and *Allaeochelys libyca* (Havlik et al., 2014), which is slightly deeper than that in *Carettochelys insculpta* (Rollot et al., 2024a; Waite, 1905; Walther, 1922). Within the quadrate fossa, the quadrate has several ridges that delineate shallow recesses and give the quadrate fossa a complex surface (Fig. 7B). Such a complex surface is also visible within the quadrate fossa of *Allaeochelys libyca* (Rollot et al., 2024b) and maybe to a lesser extent in large individuals of *Carettochelys insculpta* (Rollot et al., 2024a). Posterolateral to the quadrate fossa, the quadrate forms a slightly medially-enfolded ridge that starts from slightly above the lateral aspect of the mandibular condyle and extends dorsomedially. A similar ridge is present in *Allaeochelys libyca* (Rollot et al., 2024b) and *Carettochelys insculpta* (Rollot et al., 2024a), where it forms the posterolateral margin of the quadrate fossa. However, in DPC 7742, this ridge delineates an additional shallow, narrow fossa, where the anterior margin is marked by another low ridge that extends dorsoventrally and forms the actual entrance for the quadrate fossa (Fig. 7B). Dorsomedial to the quadrate fossa, the quadrate forms the lateral margin of the fenestra postotica and the lateral wall of the cavum acustico-jugulare. Along the posterior third of the cavum acustico-jugulare, the quadrate forms a low ridge near mid-height that delineates dorsal and ventral grooves, which we interpret as the path of the stapedial artery and lateral head vein, respectively (Fig. 7C). The dorsal groove extends anteriorly and can be followed within the cavum acustico-jugulare along the dorsal aspect of the quadrate. Anterolaterally, the groove is continuous with a shallow recess that is located just anterodorsal to the incisura columella auris (Fig. 7C). The ventromedial floor of the recess forms the posterodorsal margin of the foramen arteriomandibulare and the anterior wall of the recess forms the posterior wall of the canalis stapedio-temporalis. The medial margin of the recess anterior wall and the prootic collectively form a ridge that delineates a fenestra between the cavum acustico-jugulare ventrally and canalis stapedio-temporalis dorsally, and serves as a passage for the stapedial artery (Fig. 7C). Anteroventral to this fenestra, the recess is continuous with a groove that extends anteroventrally along the ventral surface of the prootic and passes through the anterior margin of the foramen arteriomandibulare to connect to the lateral side of the cranium. The groove is reminiscent with that identified in the same area in *Allaeochelys libyca* (Rollot et al., 2024b) and *Carettochelys insculpta* (Rollot et al., 2024a), which was shown to reflect the path of the mandibular artery, and we herein follow this interpretation for the groove in DPC 7742. The path described above for the stapedial (and mandibular) artery is very similar to that of *Allaeochelys libyca* (Rollot et al., 2024b) and *Carettochelys insculpta* (Rollot et al., 2024a), although these two differ from DPC 7742 in that they lack the medial recess of the quadrate and instead have a clearly defined groove.

**Fig. 8:**
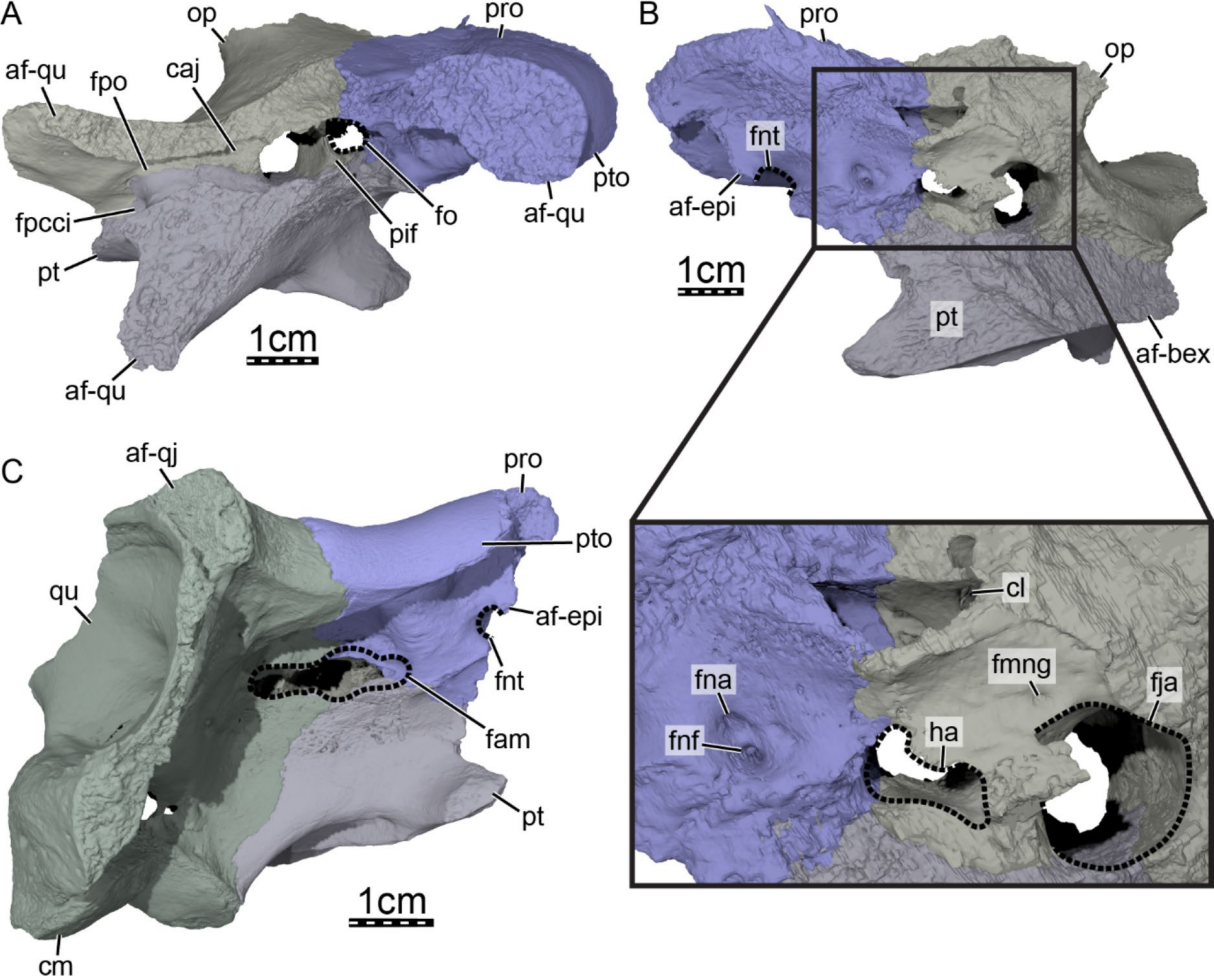
Three-dimensional renderings of the right cavum labyrinthicum and surrounding bones of *Allaeochelys meylani* sp. nov. (DPC 7742, holotype) from the Early Miocene (Burdigalian) Moghra Formation, Egypt. **A** lateral view. **B** medial view. **C** anterolateral view with addition of the quadrate. *af-bex* articular facet for basioccipital-exoccipital complex, *af-epi* articular facet for epipterygoid, *af-qj* articular facet for quadratojugal, *af-qu* articular facet for quadrate, *caj* cavum acustico-jugulare, cl cavum labyrinthicum, *cm* condylus mandibularis, *fam* foramen arteriomandibulare, *fja* foramen jugulare anterius, *fmng* foramen medialis nervi glossopharyngei, *fna* foramen nervi acustici, *fnf* foramen nervi facialis, *fnt* foramen nervi trigemini sensu stricto, *fo* foramen ovalis, *fpcci* foramen posterius canalis carotici interni, *fpo* fenestra postotica, *ha* hiatus acusticus, *op* opisthotic, *pif* processus interfenestralis of the opisthotic, *pro* prootic, *pt* pterygoid, *pto* processus trochlearis oticum, *qu* quadrate

**Prootic.** The right prootic is almost completely preserved in DPC 7742. Within the upper temporal fossa, the prootic contacts the parietal anteromedially, the supraoccipital posteromedially, the opisthotic posteriorly, and the quadrate laterally (Fig. 3A). The prootic also forms the anteromedial margin of the foramen stapedio-temporale and the medial half of the robust processus trochlearis oticum (Figs. 3A & 8A). Within the lower temporal fossa, the prootic contacts the descending process of the parietal anterodorsomedially, the pterygoid anteroventromedially, and the quadrate posterolaterally (Fig. 3B). A contact with the epipterygoid is also inferred to have occurred anteriorly (see Epipterygoid; Fig. 8B, C). The prootic forms the posterior and dorsal margin of the foramen nervi trigemini *sensu stricto* (sensu Rollot et al., 2024b) and the anterior and anterodorsal margin of the foramen arteriomandibulare (Fig. 8C). Between the foramen nervi trigemini *sensu stricto* and foramen arteriomandibulare, the prootic laterally forms a broad bulge (Fig. 8C), which visually separates the foramen nervi trigemini sensu stricto from the foramen arteriomandibulare as in *Allaeochelys libyca* (Rollot et al., 2024b). Ventrally, the prootic and pterygoid collectively form a narrow platform that extends from the foramen arteriomandibulare to the foramen nervi trigemini *sensu stricto* (Fig. 8C). As described above, we infer that the posteroventral half of the epipterygoid was likely located in that area and medially contacted the prootic and pterygoid (see Epipterygoid). The morphology of the area between the foramen arteriomandibulare and foramen nervi trigemini *sensu stricto* would therefore be greatly reminiscent of that observed in *Allaeochelys libyca* (Rollot et al., 2024b) and *Carettochelys insculpta* (Rollot et al., 2024a), in which the epipterygoid forms the anterolateral wall of an extremely constricted canalis cavernosus. This hypothesis is further supported by the absence of a canal that extends anteromedially from the anterior wall of the cavum acustico-jugulare into the braincase, by the great degree of similarity in the morphology of the cava acustico-jugulare of DPC 7742, *Allaeochelys libyca* (Rollot et al., 2024b), and *Carettochelys insculpta* (Rollot et al., 2024a), and by the location of the lateral foramen of the canalis nervus hyomandibularis proximalis (CN VII_hyo_; sensu Rollot et al., 2021), which transmits the proximal part of the hyomandibular branch towards the canalis cavernosus, just medial to the anterior margin of the foramen arteriomandibulare. The prootic forms a groove along its ventral surface, dorsomedial to the anterior margin of the foramen arteriomandibulare, which extends anteroventrally from the canalis stapedio-temporalis to the anterior margin of the foramen arteriomandibulare. This groove likely transmitted the mandibular artery (see Quadrate). The prootic also forms three shallow recesses along the anterior wall of the cavum acustico-jugulare, between the anterior margin of the fenestra ovalis and anterior margin of the foramen arteriomandibulare. The recesses are located dorsomedially, dorsally, and ventrally, and separated from one another by low ridges. The prootic forms the anterior half of the cavum labyrinthicum, the anterior half of the anterior semicircular canal, and the anterior margin of the fenestra ovalis (Fig. 8A, B). The fenestra ovalis is completely enclosed by the prootic and opisthotic (Fig. 8A), which differs from *Carettochelys insculpta*, where it is not fully surrounded by bone (Rollot et al., 2024a). The anterior half of the lateral semicircular canal is not enclosed by bone, and the prootic forms the lateral margin of a groove that contained the anterior aspect of the lateral semicircular duct. The prootic also forms the anterior margin of the hiatus acusticus (Fig. 8B), which is reduced relative to that of *Carettochelys insculpta* (Rollot et al., 2024a). In DPC 7742, the prootic and opisthotic contact one another vertically along the medial aspect of the cavum labyrinthicum and the supraoccipital dorsally, which restricts the hiatus acusticus to a small, ventral opening bordered by the prootic, opisthotic, and parabasisphenoid (Fig. 8B). In *Carettochelys insculpta*, although the size of the hiatus acusticus varies during ontogeny, a contact between the prootic and opisthotic along the medial aspect of the cavum labyrinthicum is not apparent, giving the hiatus acusticus an hourglass shape, which is bordered by the supraoccipital, prootic, opisthotic, pterygoid, and parabasisphenoid (Rollot et al., 2024a). Within the braincase of DPC 7742, the prootic forms a relatively small but deep fossa acustico-facialis, in which three foramina, two large and one small, can be identified. The large, anterodorsal foramen leads directly into the cavum labyrinthicum and transmitted the vestibulocochlear nerve (CN VIII; Fig. 8B). Posterior to that foramen, a small foramen that is barely visible in the CT scans leads into a short canal that extends from the fossa acustico-facialis to the cavum labyrinthicum. This foramen and associated canal are also inferred to have transmitted a ramus of the vestibulocochlear nerve. The more ventral large foramen leads into the canalis nervus facialis, which transmitted the facial nerve laterally through the prootic (Figs. 6B & 8B). At about mid-distance between the fossa acustico-facialis and canalis cavernosus, the canalis nervus facialis gives off a branch that extends ventrally through the prootic and pterygoid. This split is inferred to correspond to the location of the geniculate ganglion, from which the vidian and hyomandibular nerves originate. The ventral canal is the canalis pro ramo nervi vidiani, which extends ventrally through the prootic and pterygoid to join the most anterior portion of the canalis caroticus internus, transmitting the vidian nerve (Fig. 6B). The lateral canal, or canalis nervus hyomandibularis proximalis, conveyed the hyomandibular nerve laterally through the prootic from the geniculate ganglion to the canalis cavernosus (Fig. 6B). Although the facial nerve system is very similar to that of *Allaeochelys libyca* (Rollot et al., 2024b) and *Carettochelys insculpta* (Rollot et al., 2021, 2024a), two minor differences can be identified. First, the location of the geniculate ganglion in DPC7742 is slightly more medial relative to the canalis cavernosus than in *Allaeochelys libyca* and *Carettochelys insculpta*, which seems to result from a longer canalis nervus hyomandibularis proximalis. The second, minor difference is linked to the position of the junction between the canalis pro rami nervi vidiani and canalis caroticus internus. In *Allaeochelys libyca* and *Carettochelys insculpta*, the canalis pro rami nervi vidiani joins the canalis caroticus internus at about mid-length of the latter, whereas it combines with the canalis caroticus internus much more anteriorly in DPC 7742.

**Opisthotic.** The right opisthotic is almost completely preserved in DPC 7742, lacking only the posterior end of the paroccipital process. The opisthotic contacts the supraoccipital dorsomedially, the prootic anteriorly, the quadrate anterolaterally, the basioccipital-exoccipital complex ventromedially, and the pterygoid posteroventrolaterally (Fig. 3A & F). An additional contact of the paroccipital process with the squamosal was likely present posterolaterally, as in *Carettochelys insculpta*, as the articular scar is preserved. The opisthotic forms the dorsomedial margin of the fenestra postotica and the posterodorsomedial wall of the cavum acustico-jugulare (Fig. 8A). Dorsomedial to the foramen posterius canalis carotici interni, the opisthotic forms the medial margin of the foramen oropharyngeale. The paroccipital process of the opisthotic has a broad and elongate contact with the basioccipital-exoccipital complex and pterygoid ventrally, which greatly constricts the fenestra postotica medially. This ventral expansion of the paroccipital process forms the posterior wall of the wide recessus scalae tympani. The opisthotic forms the posterior half of the cavum labyrinthicum, the posterior half of the posterior semicircular canal, the posterior half of the lateral semicircular canal, the posterodorsal and posterior margins of the hiatus acusticus, and the dorsal, posterior, and ventral margins of the fenestra ovalis, which is completely surrounded by the prootic and opisthotic (Fig. 8A, B). The opisthotic ventrally forms a broad processus interfenestralis, which forms the posterior wall of the cavum labyrinthicum and the anterior wall of the recessus scalae tympani (Fig. 8A). The ventral aspect of the processus interfenestralis is developed as a massive footplate that rests on the pterygoid and also contacts the prootic anteriorly, the parabasisphenoid anteromedially, and the basioccipital-exoccipital complex posteromedially (Fig. 8A). Medial to the processus interfenestralis, the opisthotic forms a nearly vertical and slightly curved sheet of bone that forms a small portion of the braincase wall posterolaterally and, collectively with the medial edge of the processus interfenestralis, completely encloses the fenestra perilymphatica, as in adult *Carettochelys insculpta* (Rollot et al., 2024a). Along the posteroventral part of this bony sheet, the opisthotic forms a foramen that leads into a short canal that connects to the posterior part of the cavum labyrinthicum (Fig. 8B). In *Carettochelys insculpta*, this foramen corresponds to the foramen medialis nervi glossopharyngei, which transmits the glossopharyngeal nerve from the braincase to the cavum labyrinthicum (Rollot et al., 2024a). We identify the foramen in DPC 7742 as the foramen medialis nervi glossopharyngei by reference to *Carettochelys insculpta*, but note that in the latter taxon, this foramen is only present in juveniles and is absent in adults (Rollot et al., 2024a). Slightly posterolateral to the foramen medialis nervi glossopharyngei and within the cavum labyrinthicum, the opisthotic forms a small foramen internum nervi glossopharyngei. The latter foramen leads into a canal that extends posterolaterally and joins the recessus scalae tympani by means of the foramen externum nervi glossopharyngei. The foramen externum nervi glossopharyngei is located along the most dorsal aspect of the base of the processus interfenestralis, at about one third of the mediolateral width of the process. Just posteromedial to the fenestra perilymphatica, the opisthotic forms the anterior and dorsal margin of the foramen jugulare anterius (Fig. 3B).

**Supraoccipital.** The supraoccipital is heavily damaged and only its most anterior and right ventrolateral aspects are preserved in DPC 7742. The supraoccipital contacts the parietal anterodorsally, the prootic anterolaterally, the opisthotic posteroventrolaterally, and slightly contacts the basioccipital-exoccipital complex posteroventrally along the margin of the foramen magnum (Fig. 3A, C–D & F). The supraoccipital forms the dorsal margin of the cavum labyrinthicum, the posterior part of the anterior semicircular canal, and the anterior part of the posterior semicircular canal. Thus, it completely encloses the foramen aquaducti vestibuli and roofs the posterior half of the braincase. Within the braincase at the level of contact with the parietals, the supraoccipital anteriorly ends as a step-like protrusion, which corresponds to the cartilaginous rider (Werneburg et al., 2021). Although the exposure of the supraoccipital on the skull roof is moderate to minor in all carettochelyids, the preserved portions of the supraoccipital in DPC 7742 are too damaged to assess a possible exposure on the skull roof. The suture between the supraoccipital crest and parietals varies substantially between taxa and individual variation in the degree of exposure of the supraoccipital on the skull roof was described for the extant *Carettochelys insculpta* (see Joyce, 2014; Danilov et al., 2017; Joyce et al., 2018; Rollot et al., 2024a). Most of the supraoccipital crest is missing. The most posterior portion of the preserved supraoccipital nonetheless bears a short, dorsolaterally oriented process, which corresponds to the base of the expanded plate of the supraoccipital crest (Fig. 3F). The process extends posteriorly and slightly laterally, indicating that the plate was expanding mediolaterally towards the posterior to become broader posteriorly, similar to that of *Anosteira pulchra* (Joyce et al., 2018), *Allaeochelys libyca* (Rollot et al., 2024b) and adult *Carettochelys insculpta* (Rollot et al., 2024a).

**Basioccipital-exoccipital complex.** The basioccipital and exoccipitals are completely fused in DPC 7742 and we, therefore, segmented them as a single osteological unit (Fig. 3B–C & F). The right exoccipital part of the greatly thickened basioccipital-exoccipital complex is almost completely preserved and lacks only the occipital condyle, whereas the basioccipital part lacks most of its left lateral aspect. The basioccipital-exoccipital complex contacts the parabasisphenoid anteriorly along a tall contact, the supraoccipital anterodorsomedially, the opisthotic dorsally and dorsolaterally, and the pterygoid ventrolaterally (Fig. 3B–C & F). The anterior contact with the parabasisphenoid is notably higher than in *Allaeochelys libyca* (Rollot et al., 2024b) and adult *Carettochelys insculpta* (Rollot et al., 2024a). The basioccipital-exoccipital complex forms the posterolateral wall of the braincase, the lateral and ventral margins of the foramen magnum, the posteromedial wall of the recessus scalae tympani, and the posterior and ventral margins of the foramen jugulare anterius (Fig. 3C & F). Posterolateral to the foramen jugulare anterius and along the posteromedial corner of the recessus scalae tympani, the basioccipital-exoccipital complex forms a relatively large canal that extends posteroventrolaterally and exits the cranium by means of the foramen jugulare posterius. The foramen jugulare posterius is located along the posterodorsal surface of the basioccipital-exoccipital complex, directly dorsal to the posterior end of the neurapophyseal ridge (sensu Rollot et al., 2024a) and just medial to the suture with the opisthotic. The neurapophyseal ridge extends along most of the posterior surface of the basioccipital-exoccipital complex, starting dorsomedially from the suture with the supraoccipital (from which it also likely extended), and trends posteroventrolaterally on the posterior surface of the basioccipital-exoccipital complex (Fig. 3F). A similar neurapophyseal ridge is present in *Carettochelys insculpta* (Rollot et al., 2024a) and *Allaeochelys libyca* (Rollot et al., 2024b), although less pronounced in the latter taxon. The neurapophyseal ridge separates the occipital fossa ventromedially from a broad fossa dorsolaterally, whose dorsolateral half is formed by the opisthotic. These fossae are also found in *Carettochelys insculpta* but are shallower (Rollot et al., 2024a). The posterior end of the neurapophyseal ridge forms a moderately thick bony platform between the foramen jugulare posterius dorsally and the single foramen externum nervi hypoglossi. Within the braincase, posteroventromedial to the foramen jugulare anterius, the basioccipital-exoccipital complex forms two internal foramina, the foramina internum nervi hypoglossi, of which the anterior foramen is the smallest. The anterior foramen leads into a hypoglossal nerve canal that extends posteroventrally but for which the posterior part is not clearly discernible in the CT scans. The posterior foramen also leads into a hypoglossal nerve canal, which extends posteroventrolaterally and exits the skulls by means of the foramen externum nervi hypoglossi, which is located ventromedial to the posterior end of the neurapophyseal ridge. As the path of the anterior hypoglossal nerve canal was directed towards the posterior canal and only one external foramen for the hypoglossal nerve is present, we assume that the two hypoglossal nerve canals merged within the basioccipital-exoccipital complex, posteriorly forming a single hypoglossal nerve canal that joined the foramen externum nervi hypoglossi. A single external foramen for the hypoglossal nerve was also described in *Anosteira maomingensis* (Danilov et al., 2017), which contrasts with *Anosteira pulchra* (Joyce et al., 2018), *Allaeochelys libyca* (Rollot et al., 2024b) and *Carettochelys insculpta* (Rollot et al., 2024a), all of which have two external foramina for the hypoglossal nerve. The posterolateral end of the basioccipital-exoccipital complex is missing, so that the right tuberculum basioccipitale is not fully preserved. The broken area, however, forms a relatively broad rugose surface (Fig. 3F), suggesting that the basioccipital-exoccipital was somewhat more expanded posteriorly and likely formed an elongate tuberculum as in other carettochelyids (Havlik et al., 2014; Waite, 1905; Walther, 1922). The central part of the basioccipital-exoccipital complex, i.e. the area that corresponds to the basioccipital, ventrally forms a semicircular depression, which is marked by irregular ridges (Fig. 3B). A similar depression is also present in *Anosteira pulchra* (Joyce et al., 2018), *Allaeochelys libyca* (Havlik et al., 2014; Rollot et al., 2024b), and *Carettochelys insculpta* (Rollot et al., 2024a).

**Parabasisphenoid.** Most of the parabasisphenoid is not preserved in DPC 7742. We tentatively identify a small piece of bone located just ventral to the hiatus acusticus as belonging to the parabasisphenoid (Fig. 3C), as its position is similar to that of the dorsolateral aspect of the parabasisphenoid in *Allaeochelys libyca* (Rollot et al., 2024b) and *Carettochelys insculpta* (Rollot et al., 2024a). Given its extremely small size, we are only able to describe a few contacts of the putative parabasisphenoid, which are with the prootic anterolaterally, the opisthotic posterolaterally, the basioccipital-exoccipital complex posteriorly, and the pterygoid ventrolaterally. The surrounding bones indicate that the parabasisphenoid was much thicker than that of *Allaeochelys libyca* (Havlik et al., 2014; Rollot et al., 2024b) and *Carettochelys insculpta* (Rollot et al., 2024a).

**Fig. 9:**
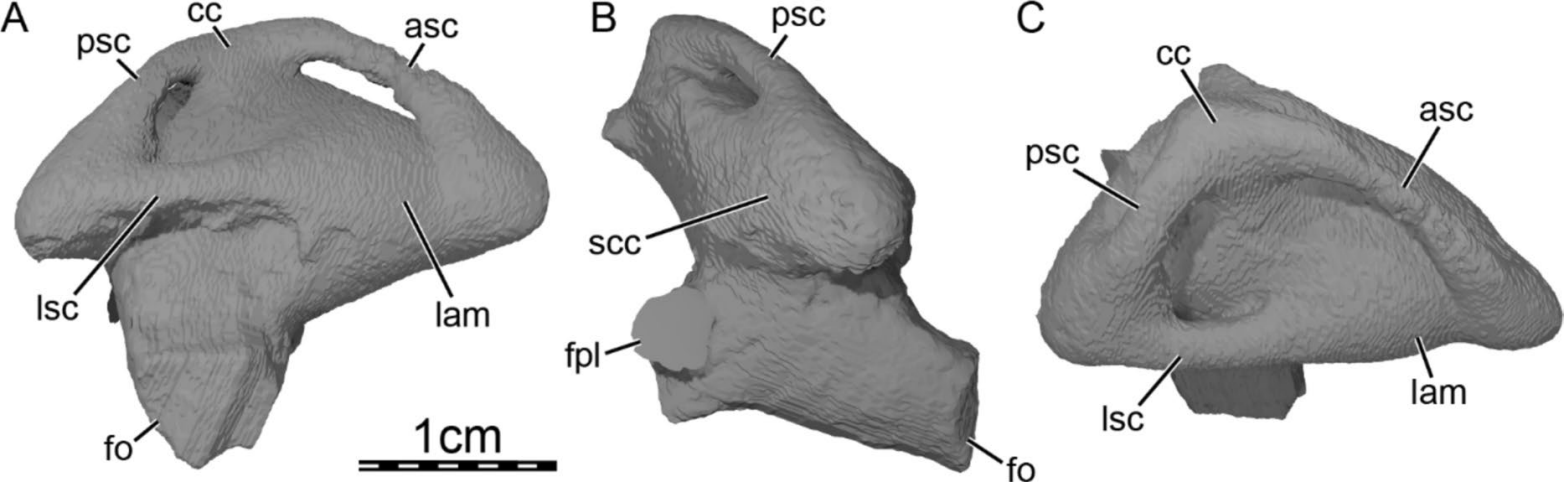
Three-dimensional renderings of the right endosseous labyrinth of *Allaeochelys meylani* sp. nov. (DPC 7742, holotype) from the Early Miocene (Burdigalian) Moghra Formation, Egypt. **A** lateral view. **B** posterior view. **C** dorsal view. *asc* anterior semicircular canal, *cc* common crus, *fo* fenestra ovalis, *fpl* fenestra perilymphatica, *lam* lateral ampulla, lsc lateral semicircular canal, *psc* posterior semicircular canal, *scc* secondary common crus

**Endosseous labyrinth.** The right endosseous labyrinth is completely preserved in DPC 7742 (Fig. 9). The semicircular canals are thick. The anterior semicircular canal is the longest of the three, and the posterior and lateral semicircular canals have approximately the same length (Fig. 9). The anterior semicircular canal anteriorly joins the vestibule at the level of the anterior ampulla. The posterior end of the posterior semicircular canal is confluent with the posterior portion of the lateral semicircular canal forming a secondary common crus (Fig. 9B; sensu Evers et al., 2019). The common crus is low as in *Allaeochelys libyca* (Rollot et al., 2024b) and *Carettochelys insculpta* (Evers et al., 2019; Rollot et al., 2024a). The dorsal embayment of the common crus is extremely subtle in DPC 7742 (Fig. 9A), which differs from *Allaeochelys libyca* (Evers et al., 2022; Rollot et al., 2024b) and *Carettochelys insculpta* (Evers et al., 2019; Rollot et al., 2024a) where the embayment is clearly defined, although slightly less pronounced in the latter. The lateral semicircular canal only forms a proper canal along its posterior portion, which is barely detached from the vestibule and creates a dorsoventral slit-like passage (Fig. 9C), as in *Allaeochelys libyca* (Rollot et al., 2024b) and *Carettochelys insculpta* (Rollot et al., 2024a). Anteriorly, the lateral semicircular canal is merged with the lateral ampulla (Fig. 9A).

#### Shell

The available shell material of *Allaeochelys meylani* sp. nov. mostly consists of isolated plates of the carapace, with the exception of one fragment. All the material was collected from the Moghra Formation but is not directly affiliated with the available cranium. We here describe and figure elements that are either complete or only suffer minor damage, allowing their identification with relative ease (pictures of additional, fragmentary material are available upon request). All plates exhibit the typical ornamentation of the *Allaeochelys*/*Carettochelys* lineage, consisting of thick ridges separated by equally sized grooves. Much like the cranium, their most striking feature is their remarkably large size.

**Fig. 10:**
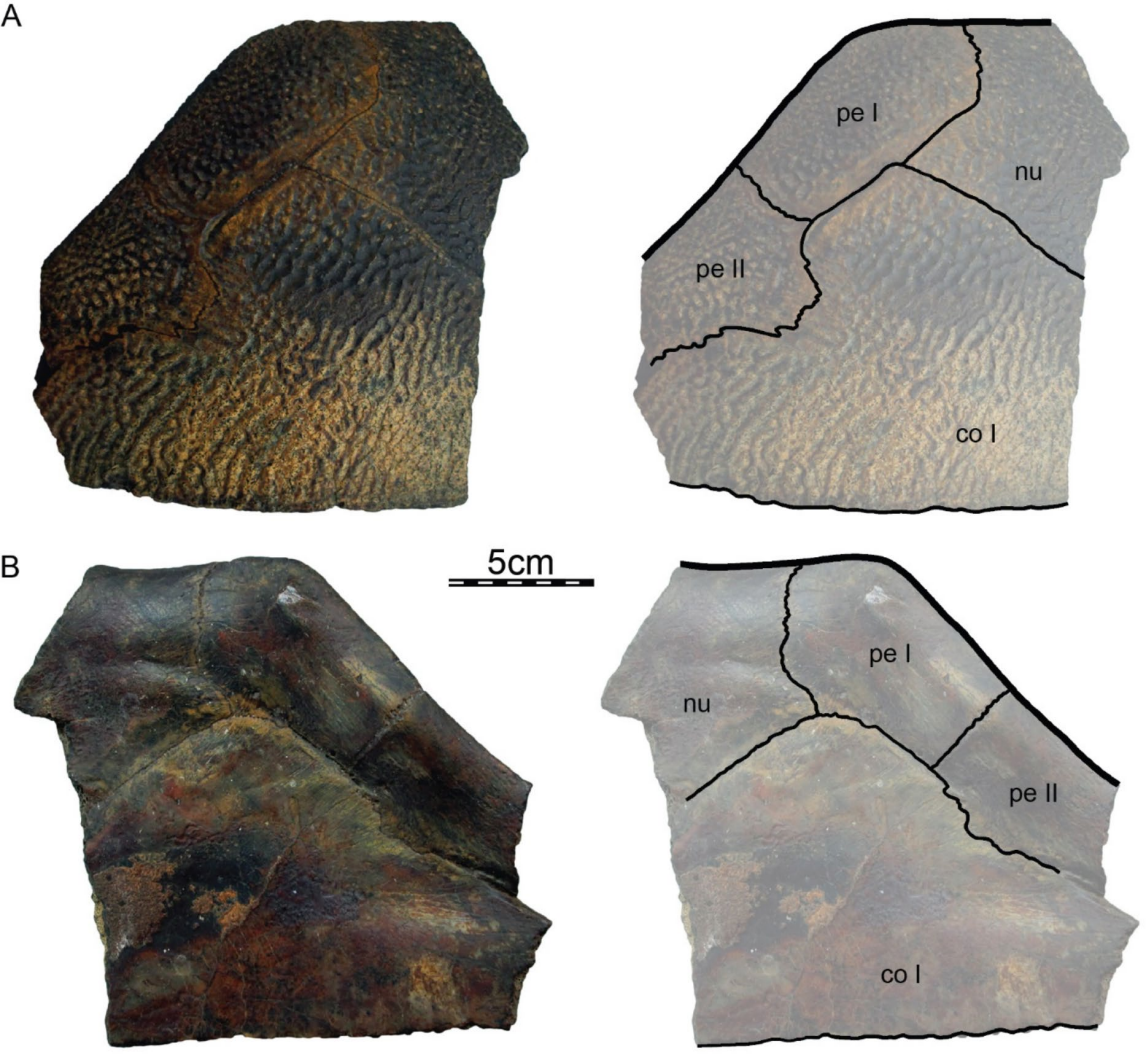
Anterolateral portion of carapace of *Allaeochelys meylani* sp. nov. (CGM 67151) from the Early Miocene (Burdigalian) Moghra Formation, Egypt. **A** picture and drawing in dorsal view. **B** picture and drawing in ventral view. *co I* costal I, *nu* nuchal, *pe I* peripheral I, *pe II* peripheral II

**Nuchal.** We identify two elements as nuchals. CGM 67151 is a large carapace fragment that includes a partial nuchal, left peripherals I and II, and left costal I, and the preservation and sutural contacts between the bones makes their identification clear (Fig. 10). Most of the nuchal is not preserved, but the lateral portion of the element shows that the nuchal forms the anterior margin of the carapace and contacts peripheral I laterally and costal I posterolaterally (Fig. 10).

**Fig. 11:**
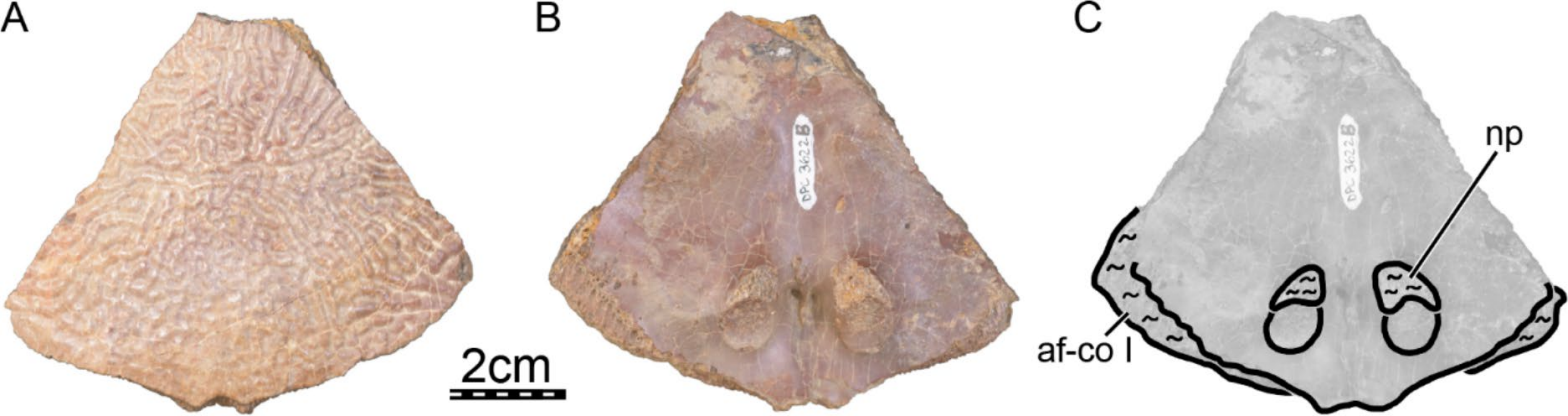
Nuchal of *Allaeochelys meylani* sp. nov. (DPC 3622) from the Early Miocene (Burdigalian) Moghra Formation, Egypt. **A** dorsal view. **B** ventral view. **C** drawing in ventral view. *af-co I* articular facet for costal I, *np* nuchal process

The second element that can confidently be identified as a nuchal is DPC 3622 (Fig. 11). This fossil is not complete, lacking its anterior and lateral margins (Fig. 11A, B). The approximate length and width of the preserved part are 7 cm and 8 cm, respectively. The posterior margin of DPC 3622 is intact and preserves the posterolateral articulation facet with costal I and the concave articulation facet for neural I (Fig. 11C). The narrow nature of this facet suggests that the neurals were narrow and elongate, as in *Carettochelys insculpta*. The nuchal forms a pair of processes in the posterior quarter on its ventral surface (Fig. 11B, C), which are typical of pan-carettochelyids (Joyce, 2014). The base of the nuchal processes is expanded posteriorly, forming a shallow, cup-like depression (Fig. 11B, C).

**Peripherals.** We identify five elements as peripherals. CGM 67151 preserves the left peripherals I and II (Fig. 10). The left peripheral I is complete and approximately 7 cm long, and contacts the nuchal medially, left costal I posteromedially and left peripheral II posterolaterally (Fig. 10). The left peripheral II lacks its posterolateral end, but the length of the full element can be estimated to be about 6–7 cm. The left peripheral II contacts left peripheral I anteromedially and left costal I medially (Fig. 10). The element has a short but broad medial process that protrudes into left costal I (Fig. 10). The process is distinctly visible on the dorsal side of the carapace but to a much lesser extent on the visceral side (Fig. 10).

**Fig. 12:**
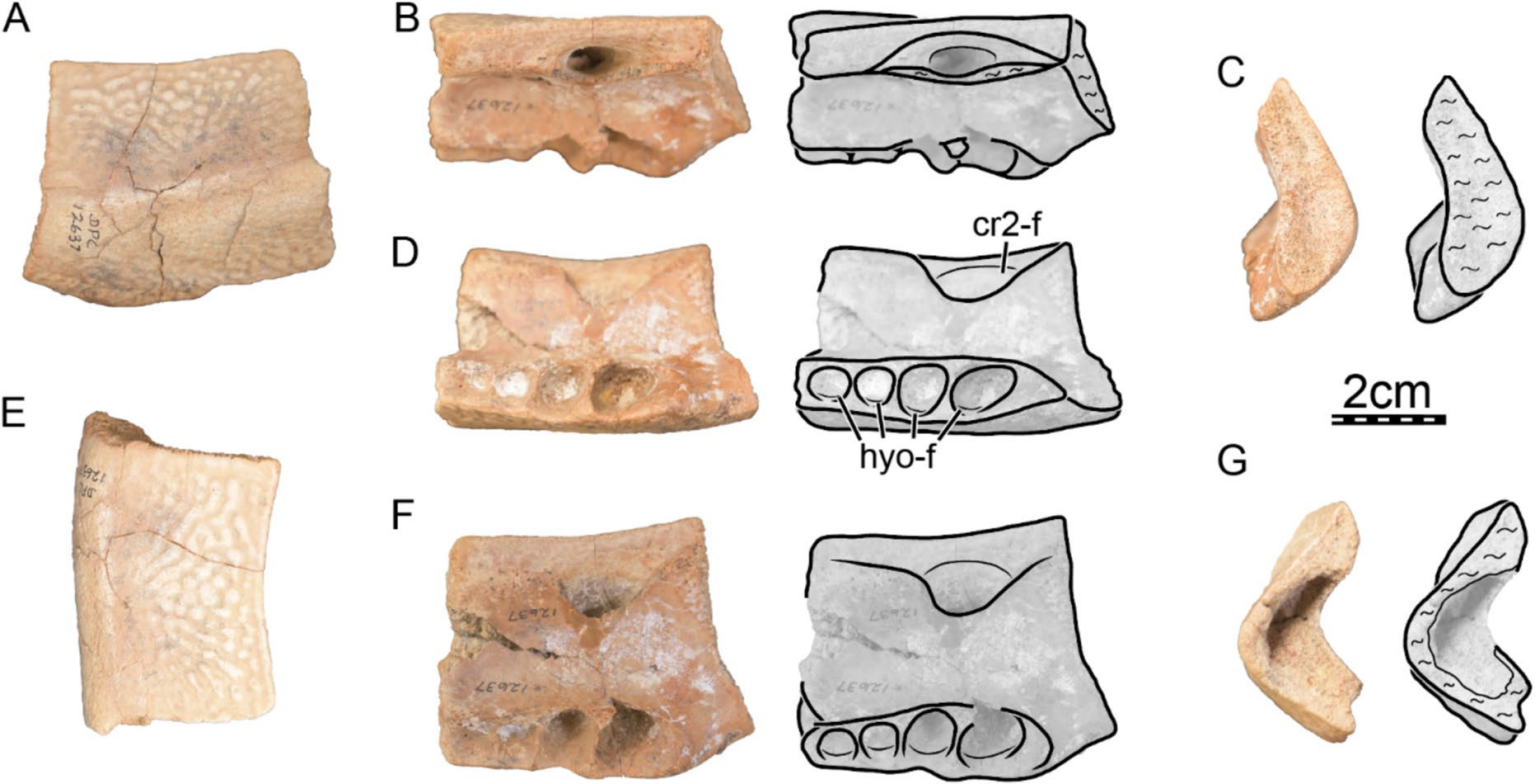
Left peripheral IV of *Allaeochelys meylani* sp. nov. (DPC 12637) from the Early Miocene (Burdigalian) Moghra Formation, Egypt. **A** lateral view. **B** picture and drawing in dorsomedial view. **C** picture and drawing in anterior view. **D** picture and drawing in ventromedial view. **E** dorsolateral view. **F** picture and drawing in medial view. **G** picture and drawing in posterior view. *cr2-f* facet for costal rib II, *hyo-f* facet for hyoplastron

DPC 12637 can be identified as a left peripheral IV, which is approximately 6 cm long (Fig. 12). The element has a C shape in cross section and forms four facets for articulation with the hyoplastron along its ventromedial side (Fig. 12C–D & F–G). The articulation facets for the hyoplastron are large, with a slight, gradual decrease in size posteriorly (Fig. 12D & F). Anterior to the most anterior facet, the ventromedial margin of the peripheral is delineated by a smooth margin that shows the anterior end of the bridge (Fig. 12D). This allows identification as the peripheral IV by comparison to *Carettochelys insculpta*. Dorsomedially, the peripheral forms an articulation facet for the left costal rib II (Fig. 12D & F). The external surface of the peripheral is crossed by a low ridge that extends anteroposteriorly at about mid-height of the element to form the lateral margin of the shell, and this morphology is absent in *Carettochelys insculpta* (Fig. 12A).

**Fig. 13:**
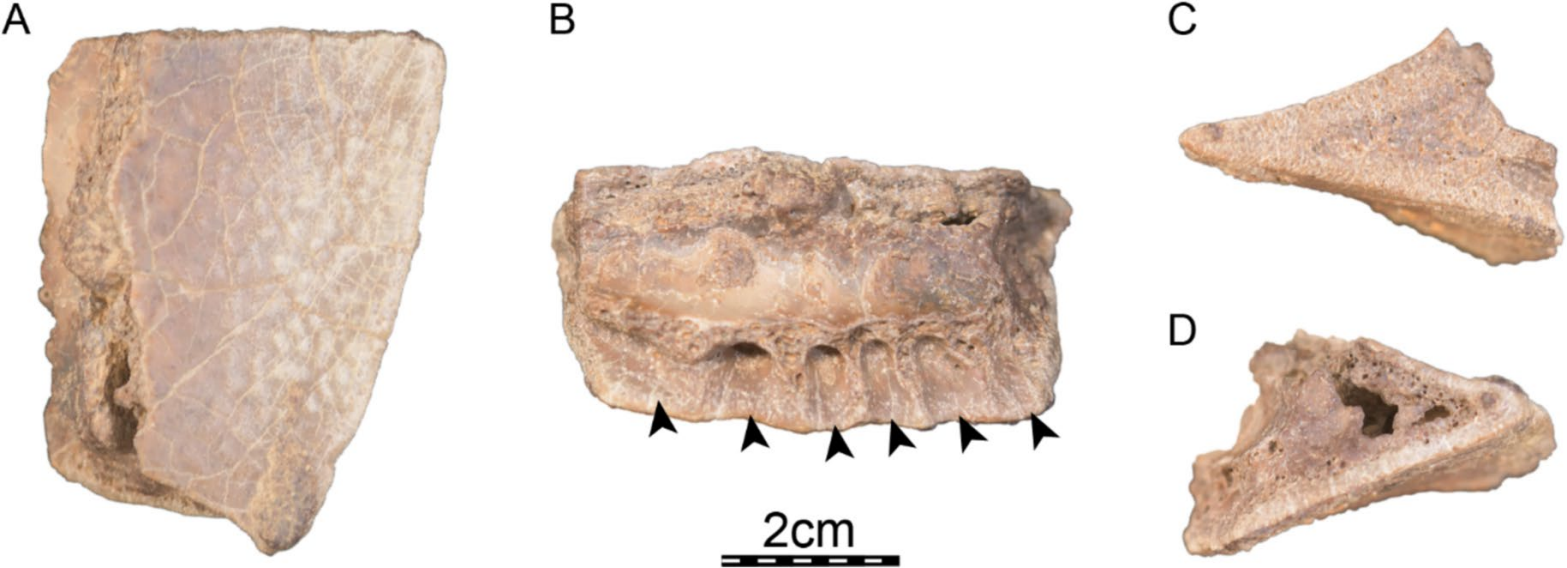
Right peripheral VI of *Allaeochelys meylani* sp. nov. (DPC 12585) from the Early Miocene (Burdigalian) Moghra Formation, Egypt. **A** dorsal view. **B** medial view. **C** anterior view. **D** posterior view. Black arrowheads indicate the facets for articulation with the hypoplastron

DPC 12585 is approximately 6 cm long, V-shaped in cross section, possesses 6 facets ventromedially for articulation with either the hyo- or hypoplastron, and has a straight medial margin (Fig. 13). As the element lacks a smooth margin anterior to the hyo-hypoplastral facets (which is diagnostic for peripheral IV), a smooth margin posterior to the hyo-hypoplastral facets (which is diagnostic for peripheral VII), and lacks a medial process that protrudes medially into the hyo-hypoplastral suture (typical of peripheral V), the morphology of the element is consistent with that of a right peripheral VI in *Carettochelys insculpta*, and is identified as such herein. The dorsomedial part of the peripheral, i.e. the area that forms the articulation facet for the right costal IV, is missing (Fig. 13). The peripheral is dorsoventrally flatter than peripheral IV.

**Fig. 14:**
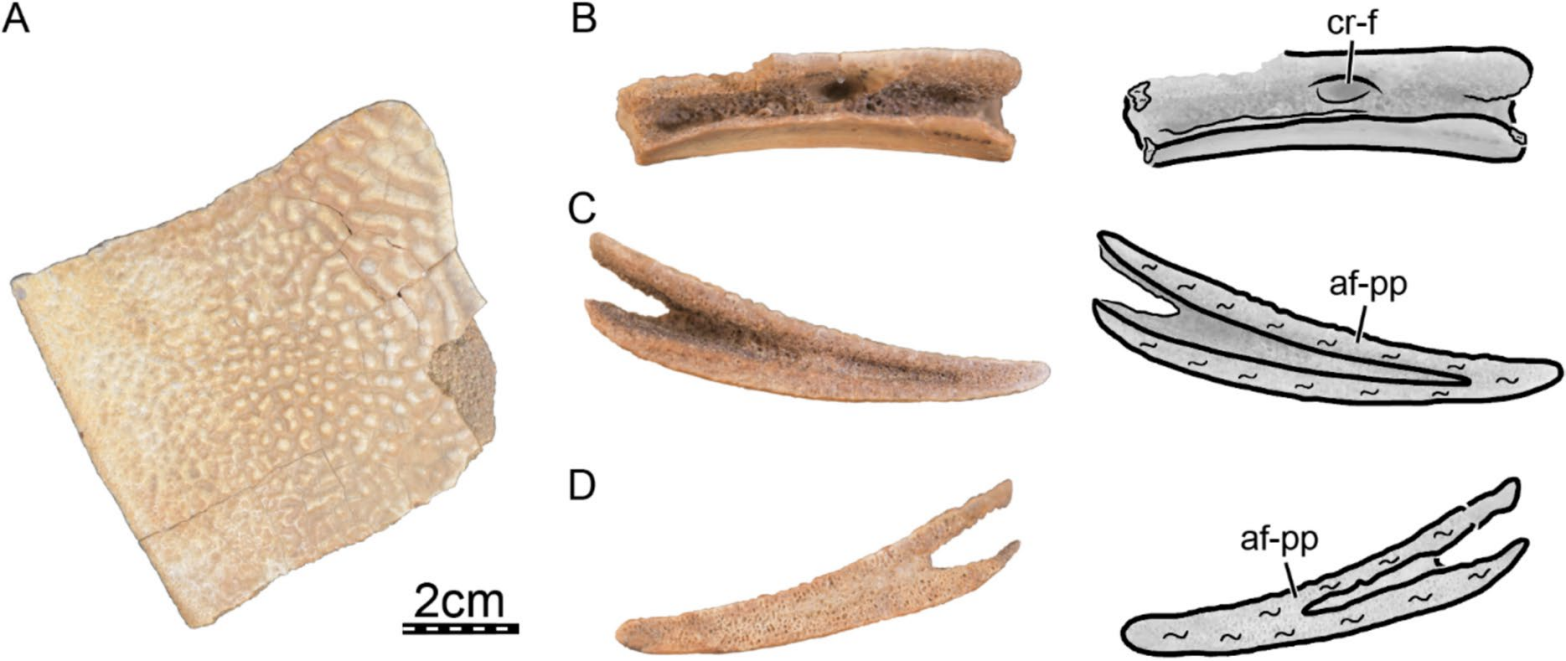
Posterior peripheral of *Allaeochelys meylani* sp. nov. (DPC 6436A) from the Early Miocene (Burdigalian) Moghra Formation, Egypt. **A** dorsal view. **B** picture and drawing in medial view. **C** picture and drawing in anterior view. **D** picture and drawing in posterior view. *af-pp* articular facet for posterior peripheral

The last element that we identify as a peripheral is DPC 6436 (Fig. 14). The element is nearly intact and measures approximately 7 cm in length and 8 cm in width. The large size, flatness, and square shape of the element have affinities with a posterior peripheral. However, a dorsomedial portion of the element and most of the ventromedial lip are missing, which precludes assessment of its exact morphology and, consequently, prevents us from identifying it precisely as a peripheral VIII, IX, or X. Dorsomedially, at about mid-length, the peripheral has an articulation facet for a costal rib end (Fig. 14B).

**Costals.** CGM 67151 preserves most of a left costal I. It is a large element and almost as long as broad that only lacks a small portion of its medial and lateral sides (Fig. 10). The left costal I contacts the nuchal anteriorly, left peripheral I anterolaterally, and left peripheral II laterally (Fig. 10). A lateral contact with left peripheral III can also be reasonably inferred, as most of left peripheral II is preserved and it barely extends beyond about mid-length of left costal I. The lateral margin of left costal I is concave as a result of the medial process of left peripheral II that protrudes into the costal.

**Fig. 15:**
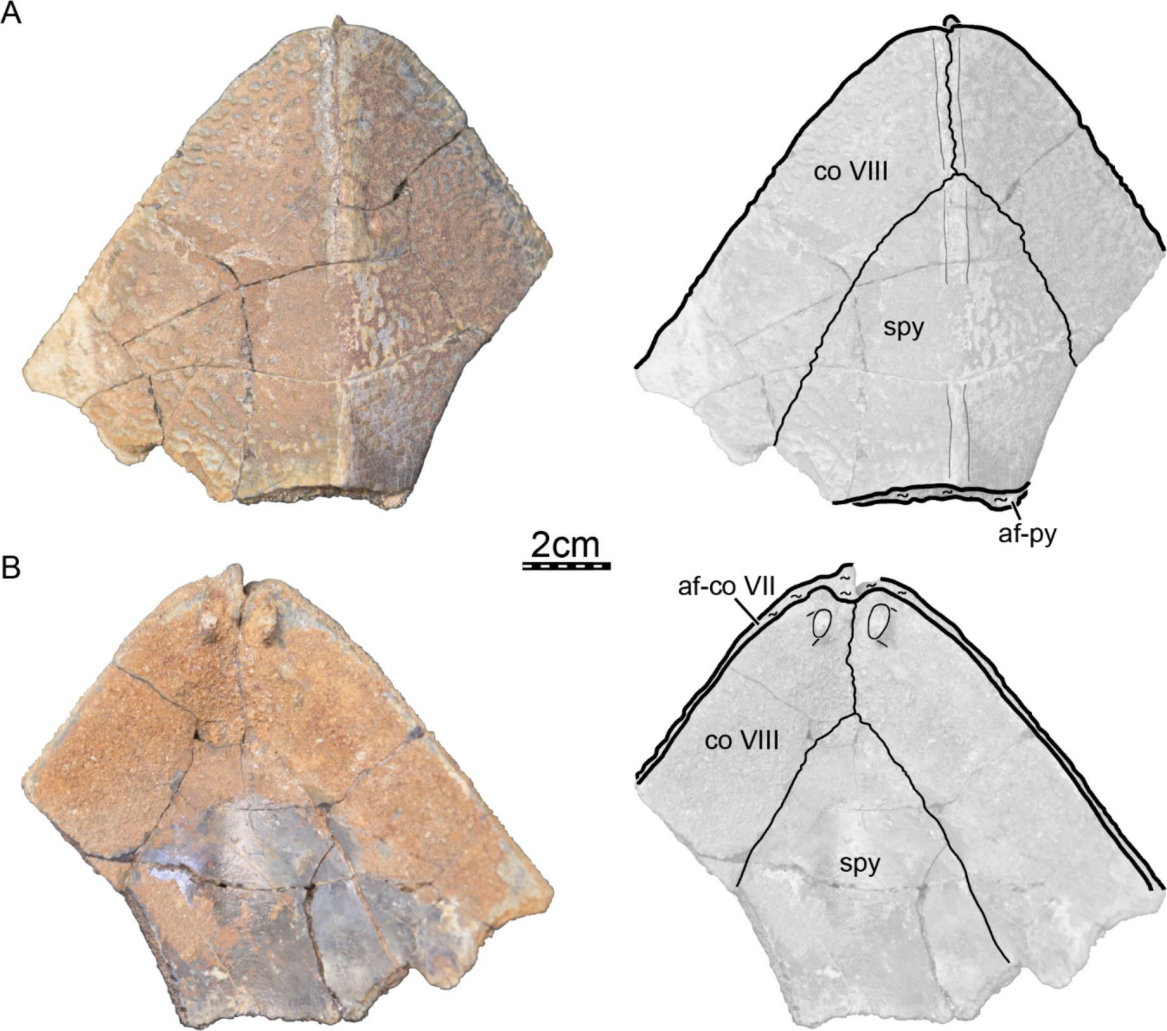
Costals VIII and suprapygal of *Allaeochelys meylani* sp. nov. (DPC 14555) from the Early Miocene (Burdigalian) Moghra Formation, Egypt. **A** picture and drawing in dorsal view. **B** picture and drawing in ventral view. *af-co VII* articular facet for costal VII, *af-py* articular facet for pygal, *co VIII* costal VIII, *spy* suprapygal

DPC 14555 is a carapacial fragment including the bilateral costals VIII and the suprapygal (Fig. 15). The left costal VIII only lacks the most lateral aspect that formed the associated costal rib, whereas the right costal VIII only preserves its medial half (Fig. 15). The preserved contacts of costal VIII are with its counterpart medially and the suprapygal posteromedially (Fig. 15). By reference to other pan-carettochelyids, we also infer that costal VIII had a contact with costal VII anteriorly and peripheral X posterolaterally (Adrian et al., 2020; Broin, 1977; Carbot-Chanona et al., 2020; Clark, 1932; Dollo, 1886; Harrassowitz, 1922; Hay, 1906; Nessov, 1977; Ramsay, 1887; Tong et al., 2010; Waite, 1905). A contact with the posteromedial edge of peripheral IX might have occurred as well, as such a contact was reported in *Kizylkumemys schultzi* (Nessov, 1977), *Anosteira ornata* (Hay, 1906), *Allaeochelys delheidi* (Dollo, 1886), *Allaeochelys crassesculpta* (Harrassowitz, 1922), *Allaeochelys parayrei* (Broin, 1977), *Allaeochelys liliae* (Carbot-Chanona et al., 2020), and some specimens of *Carettochelys insculpta* (Joyce, 2014). The anterior margin of costals VIII collectively forms a rounded and regular, uninterrupted margin, indicating that an anteromedial contact with a neural is absent and that DPC 14555 likely had no more than 7 neurals (Fig. 15A), as in other pan-carettochelyids (Adrian et al., 2020; Broin, 1977; Carbot-Chanona et al., 2020; Clark, 1932; Harrassowitz, 1922; Hay, 1906; Nessov, 1977; Ramsay, 1887; Tong et al., 2010; Waite, 1905; Zangerl, 1947). Collectively with the suprapygal, costals VIII form a low keel along the dorsal midline of the carapace (Fig. 14A), consistent with most pan-carettochelyids (Adrian et al., 2020; Broin, 1977; Carbot-Chanona et al., 2020; Cheng, 1961; Chow & Liu, 1955; Clark, 1932; Harrassowitz, 1922; Hutchison et al., 2004; Nessov, 1977; Tong et al., 2010; Waite, 1905; Zangerl, 1947), but not *Kizylkumemys khoratensis* (Tong et al., 2005).

**Suprapygal.** A suprapygal is part of DPC 14555, along with the two costals VIII described above. The suprapygal is relatively complete and lacks its posterolateral aspect on both sides (Fig. 15). It is approximately 7 cm long along the midline and contacts costal VIII laterally (Fig. 15). The posterior surface of the suprapygal forms an intact, sutural surface for the articulation with the pygal for most of its width (Fig. 15A). Although not preserved, the posterolateral aspect of the suprapygal likely had a contact with peripheral X, as in other pan-carettochelyids (Broin, 1977; Carbot-Chanona et al., 2020; Clark, 1932; Dollo, 1886; Harrassowitz, 1922; Hay, 1906; Nessov, 1977; Waite, 1905). Along its dorsal surface, the suprapygal forms a low keel that extends anteroposteriorly along the midline of the carapace as in most pan-carettochelyids (Fig. 15A; Adrian et al., 2020; Broin, 1977; Carbot-Chanona et al., 2020; Cheng, 1961; Chow & Liu, 1955; Clark, 1932; Harrassowitz, 1922; Hutchison et al., 2004; Nessov, 1977; Tong et al., 2010; Waite, 1905; Zangerl, 1947), but not *Kizylkumemys khoratensis* (Tong et al., 2005).

**Fig. 16:**
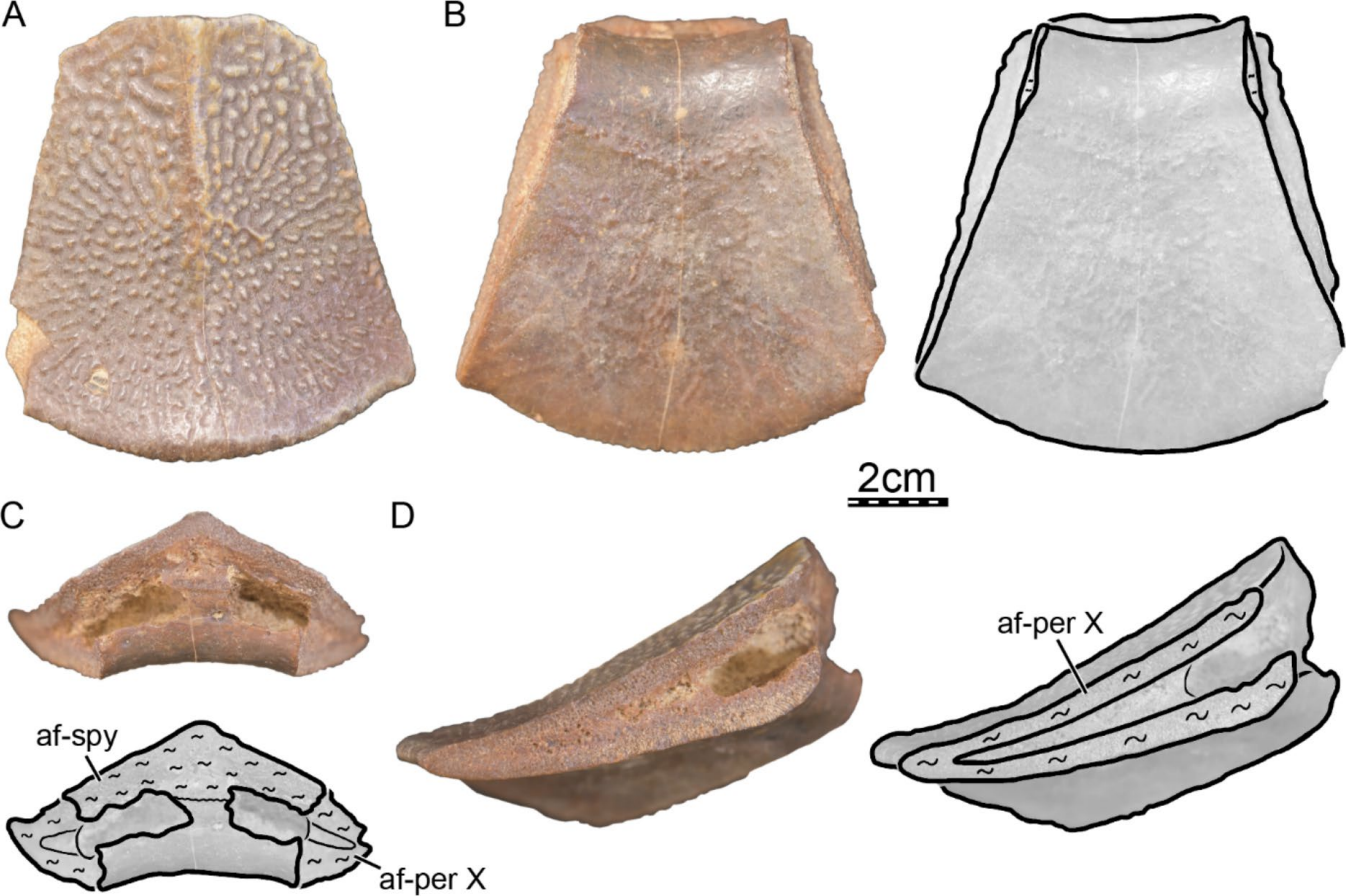
Pygal of *Allaeochelys meylani* sp. nov. (DPC 7741) from the Early Miocene (Burdigalian) Moghra Formation, Egypt. **A** dorsal view. **B** picture and drawing in ventral view. **C** picture and drawing in anterior view. **D** picture and drawing in right lateral view. *af-per X* articular facet for peripheral X, *af-spy* articular facet for suprapygal

**Pygal.** DPC 7741 is identified as a pygal, which is almost complete and only lacks its left posterolateral edge (Fig. 16). This element is approximately 9 cm long and 8 cm wide. The lateral surface of the pygal forms an articulation facet that is deep and V-shaped and serves as a contact area with peripheral X (Fig. 16D). The dorsal part of the suture is continuous towards the anterior surface of the pygal, forming an articulation facet for contact with the suprapygal anteriorly (Fig. 16C). The ventral part of the pygal-peripheral X suture is limited to the lateral surface of the pygal, so that the anteroventral surface (i.e., visceral side) of the pygal is smooth and forms a thick lip (Fig. 16B–D), which in pan-carettochelyids extends laterally on the posterior peripherals (Joyce, 2014). Along its dorsal surface, the pygal forms a low keel that extends along its approximately anterior half (Fig. 16A).

**Fig. 17:**
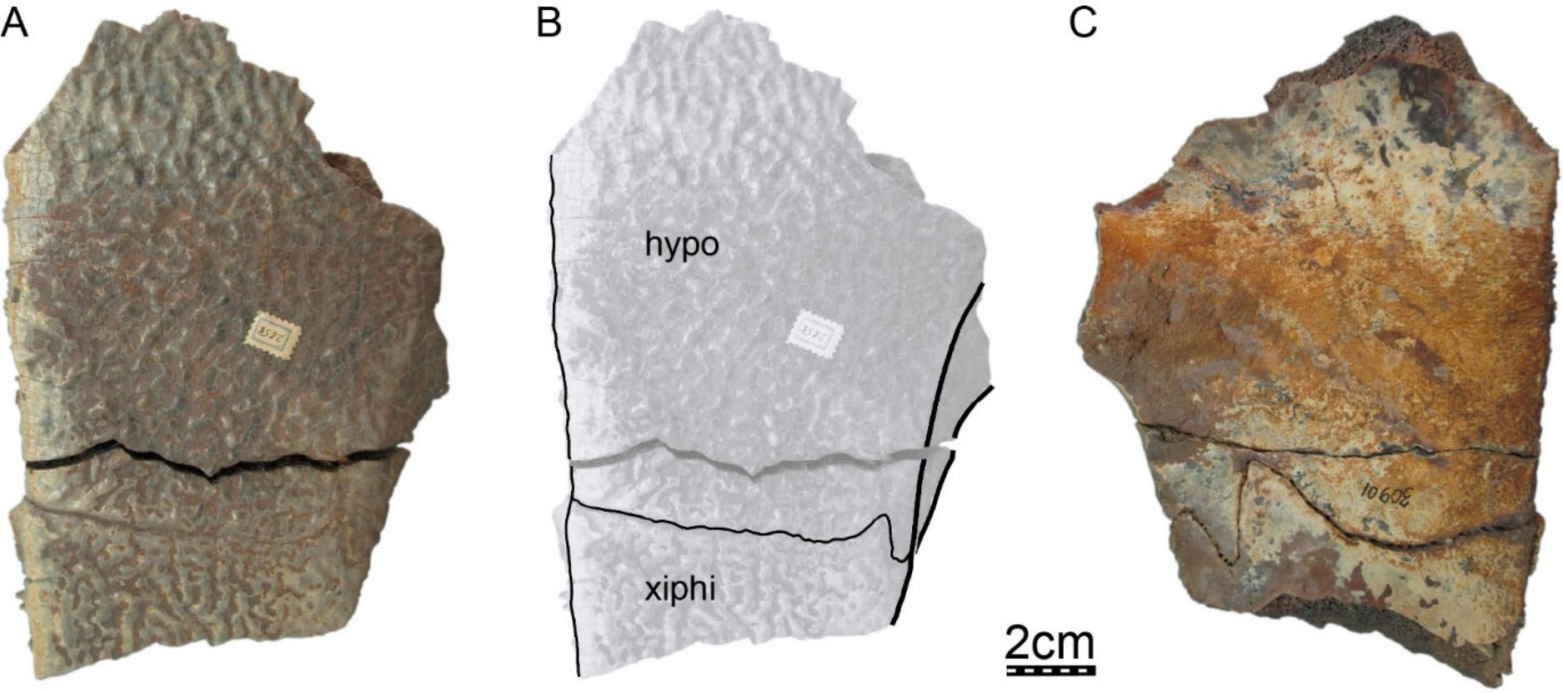
Partial left hypoplastron and left xiphiplastron of *Allaeochelys meylani* sp. nov. (CGM 67140) from the Early Miocene (Burdigalian) Moghra Formation, Egypt. **A** ventral view. **B** drawing in ventral view. **C** dorsal view. *hypo* hypoplastron, *xiphi* xiphiplastron

**Hypoplastron.** CGM 67140 is a left plastral fragment that consists of a partial hypoplastron and a partial xiphiplastron (Fig. 17). The element mostly preserves the medial half of the left hypoplastron, showing the medial suture with its counterpart and the medial margin of the inguinal notch. The hypoplastron posteriorly contacts the xiphiplastron along a straight suture for most of its width, but it has a well-defined sinusoid shape at its lateral end on the visceral side of the element (Fig. 17C). The preserved, medial portion of the hypoplastron is notably broad, reminiscent of the hypoplastron of other *Allaeochelys* taxa and also that of *Carettochelys insculpta* (Joyce, 2014).

**Xiphiplastron.** CGM 67140 includes a partial left xiphiplastron that represents about the anterior third of the element (Fig. 17). The medial side of the left xiphiplastron forms a sutural surface for contact with its counterpart. The xiphiplastron anteriorly contacts the hypoplastron. The hypo-xiphiplastron suture is straight for most of its width but forms a well-defined sinusoid line laterally (Fig. 17).

Trionychidae Bell, 1828

*Trionyx* Geoffroy Saint-Hilaire, 1809

*Trionyx* sp.

### Description

**Fig. 18:**
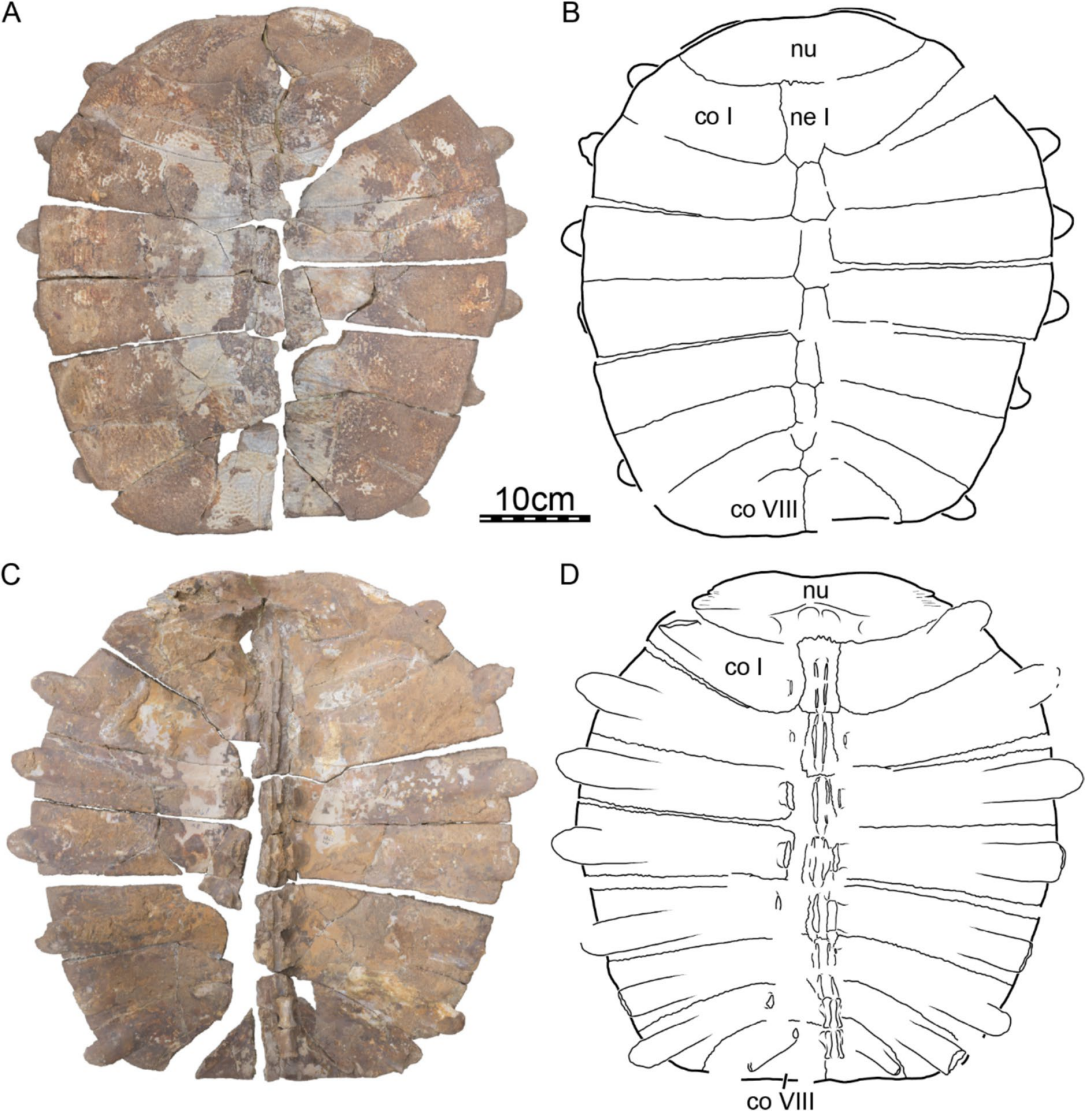
Carapace of *Trionyx* sp. (DPC 7789) from the Early Miocene (Burdigalian) Moghra Formation, Egypt. **A** picture in dorsal view. **B** drawing of carapace in dorsal view. **C** picture in ventral view. **D** drawing of carapace in ventral view. *co I* costal I, *co VIII* costal VIII, *ne I* neural I, nu nuchal

**Fig. 19:**
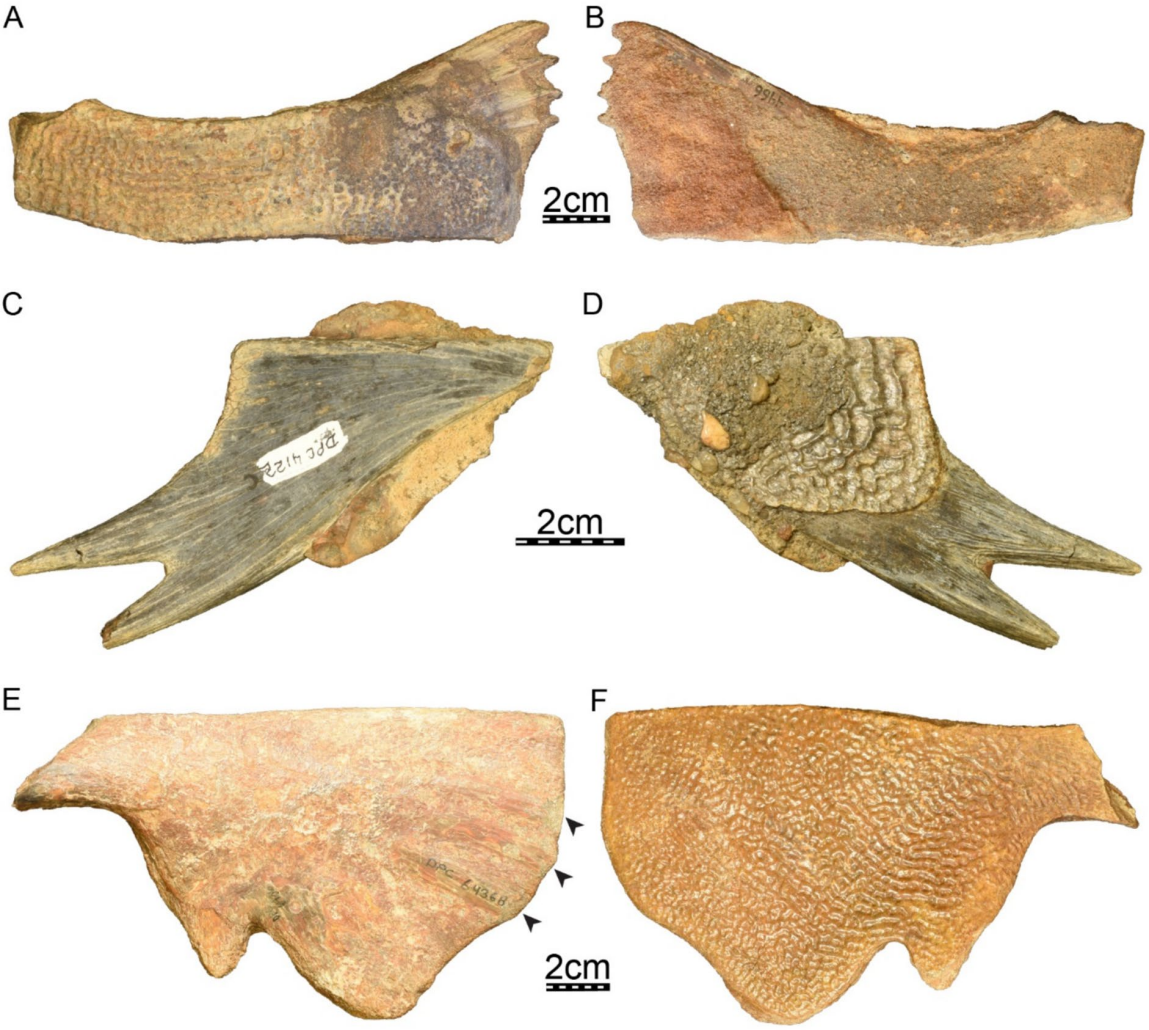
Plastral elements of *Trionyx* sp. from the Early Miocene (Burdigalian) Moghra Formation, Egypt. **A** right hyoplastron (DPC 4466) in ventral view. **B** right hyoplastron (DPC 4466) in dorsal view. **C** posterolateral part of a left hypoplastron (DPC 4122) in dorsal view. **D** posterolateral part of left hypoplastron (DPC 4122) in ventral view. **E** left hypoplastron (DPC 6436B) in dorsal view. **F** left hypoplastron (DPC 6436B) in ventral view. Black arrowheads indicate the medial and posteromedial processes of the hypoplastron

**General comments.** DPC 7789 consists of six fragments that together represent a nearly complete carapace (Fig. 18). The carapace is round and consists of a single unpaired nuchal, seven neurals, and eight pairs of costals (Fig. 18). As in all trionychids, peripherals, suprapygals, and pygals are absent (Meylan, 1987). Although many trionychid plastral elements are available in the collections of the DPC, most of these are fragmentary. We, therefore, only formally figure and describe three elements that bear sufficient information with regards to the taxonomic identity of the remains (see Fig. 19). Those elements are a nearly complete right hyoplastron (DPC 4466), a partial left hypoplastron (DPC 4122), and a nearly complete left hypoplastron (DPC 6436). All metaplastic portions of the shell bones are covered by a distinct sculpturing that consists of pits separated by vermiculated ridges, which tend to line up towards the margins. Scutes and their sulci are universally absent.

**Nuchal.** The nuchal forms the most anterior aspect of the shell and the anterior shell margin (Fig. 18). The nuchal is anteroposteriorly deep, approximately three times wider than long, and contacts costal I posterolaterally and neural I posteriorly (Fig. 18). The lateral half of the contact between the nuchal and costal I is strongly curved towards the anterior (Fig. 18). The costiform process forms wing-like processes that are not fully covered dorsally by the metaplastic ossification of the bone and slightly expand over the margin of the carapace anteriorly and anterolaterally (Fig. 18A, B). In the middle of its posterior half, the nuchal ventrally forms paired depressions that provided space for the articulation of the eighth cervical vertebrae with the thoracic vertebral column (Fig. 18C, D). Suprascapular fontanelles are absent (Fig. 18A, B).

**Costals.** Costal I is curved anteriorly, relatively reduced in mediolateral width, and follows the shape of the posterior suture of the nuchal while tapering slightly laterally (Fig. 18). Costal II is slightly curved anteriorly, albeit less than costal I, and laterally expanded mediolaterally (Fig. 18). Costals III and IV are almost straight, whereas costals V, VI, and VII are curved posteriorly with the degree of curvature progressively increasing posteriorly (Fig. 18). Costal VIII is a roughly triangular element that forms the posterior margin of the shell (Fig. 18). Costal I only contacts the first neural, whereas costals II to IV contact neurals I–II, II–III, and III–IV, respectively (Fig. 18A, B). Left costal V contacts neurals IV, V and VI, but its right counterpart meets only neurals IV and V (Fig. 18A, B). Conversely, while left costal VI contacts neurals IV–VII, right costal VI contacts neurals V, VI, and VII. Costal VII only contacts neural VII (Fig. 18A, B). Costals VII also have a medial contact with one another along their posterior half, posterior to neural VII, while costals VIII medially contact one another for their entire length (Fig. 18A, B). The right costal VII also contacts the left costal VIII posteromedially (Fig. 18A, B). Although some of the lateral ends of the ribs are damaged, all of them extended beyond the metaplastically-ossified portion of the costals as wide, convex projections with the exception of the lateral end of costal rib VIII (Fig. 18).

**Neurals.** DPC 7789 includes seven neurals that form an uninterrupted series (Fig. 18A, B). Neural I is hexagonal with straight lateral margins (Fig. 18A, B). Neurals II to IV are hexagonal with lateral margins that are slightly oriented posterolaterally and short posterolateral sides (Fig. 18A, B). Neurals V and VI are pentagonal and asymmetrical, thus jointly forming the neural reversal (Fig. 18A, B). Neural VII is the shortest of all neurals, heptagonal in shape, has five contacts, and prevents costals VII from contacting one another for their anterior half (Fig. 18A, B). Neurals I to IV each contact two pairs of costals: costals I–II, II–III, III–IV, and IV–V respectively (Fig. 18A, B). Neural V contacts costal V on the left side but costals V and VI on the right side, whereas neural VI contacts costals V and VI on the left side but only costal VI on the right side (Fig. 18A, B). Neural VII contacts costal VI and VII (Fig. 18A, B).

**Hyoplastron.** DPC 4466 is a nearly complete right hyoplastron that only lacks its anterolateral tip, which prevents us from determining the number of anterolateral processes (Fig. 19A, B). The hyoplastron is approximately four times wider than long (Fig. 19A, B). The hyoplastral callosity is well developed and covers most of the element with the exception of the anteromedial part, where at least four finger-like processes are preserved (Fig. 19A). The anterior margin of the hyoplastral callosity protrudes slightly into the axillary notch formed by the processes and is nearly straight (Fig. 19A). The hyoplastron forms a suture along its posterior margin for articulation with the hypoplastron (Fig. 19A, B). Medially, the callosity forms a smooth margin, which shows that the bilateral hyoplastra were not medially sutured to one another (Fig. 19A).

**Hypoplastron.** DPC 4122 and DPC 6436 together preserve nearly the entire morphology of a left hypoplastron, which is broad and elongate (Fig. 19C–F). The hypoplastral callosity is well developed and medially covers most of the medial processes (Fig. 19E, F). Posterolaterally, the hypoplastron forms two large lateral processes that protrude beyond the margin of the callosity (Fig. 19C, D). The anterior margin of the hypoplastron is nearly straight and forms a sutural surface for contact with the hyoplastron (Fig. 19E, F). Medially, the margin of the hypoplastral callosity is smooth, suggesting that a sutural contact with its counterpart is absent (Fig. 19F). Posteriorly, the hypoplastron forms a deep notch for the articulation with one of the anterolateral processes of the xiphiplastron (Fig. 19E, F). Although posteromedial processes are not apparent beyond the hypoplastral callosity, the ventral surface of the hypoplastron is crossed by at least three processes that suggest the former presence of an uneven medial comb (Fig. 19E).

## Discussion and conclusions

### Alpha taxonomy – *Allaeochelys meylani* sp. nov.

*Carettochelyidae* is a relatively poorly diversified clade of turtles with only 18 valid species recognized from the Early Cretaceous to Neogene (Carbot-Chanona et al., 2020; Godinot et al., 2018; Havlik et al., 2014; Hutchison & Westgate, 2024; Joyce, 2014; Tong et al., 2005; White et al., 2023). The Paleogene is particularly rich with 12 valid species, while only two are known from the Cretaceous and three from the Neogene. Although the *Allaeochelys*/*Carettochelys* lineage had previously been documented from the Neogene by isolated and fragmented remains (Dacqué, 1912; Hirayama, 1992; Thomas et al., 1981; de Lapparent de Broin, 2000; Joyce et al., 2004; Glaessner, 1942), more complete material that is diagnostic to the species level was only described over the course of the last decade, in particular *Carettochelys niahensis* from the Late Oligocene–Early Miocene of Malaysia (White et al., 2023), *Allaeochelys liliae* from the Early Miocene (Aquitanian) of Mexico (Carbot-Chanona et al., 2020), and *Allaeochelys libyca* from the Middle Miocene (Langhian) of Libya (Havlik et al., 2014). *Allaeochelys meylani* sp. nov., therefore, represents the fourth carettochelyid taxon recognized in the Miocene, and is also the oldest known carettochelyid taxon in Afro-Arabia. Although remains coeval to that of *Allaeochelys meylani* sp. nov. were reported from the Burdigalian of Saudi Arabia (see Thomas et al., 1981), these are not associated with any description, figure, illustration, and specimen number, which prevents confirming the taxonomic identity and nature of the material. We, therefore, dismiss this material herein from our paleobiogeographical considerations.

The carettochelyid cranium described in the present contribution can confidently be identified as a carettochelyine (= *Allaeochelys*/*Carettochelys* lineage) by the presence of a contact between the maxilla and quadratojugal, a foramen posterius canalis carotici interni distant from the posterior border of the parabasisphenoid, and a deep quadrate fossa. The cranium is furthermore sufficiently preserved to allow recognition of a new *Allaeochelys* species (see further below), but this assertion is also supported by temporal arguments. Indeed, the present material from the Moghra Formation is Burdigalian in age and, therefore, fills a temporal gap between previously reported taxa from the Aquitanian (*Allaeochelys liliae* and, likely, *Carettochelys niahensis*) and the Langhian (*Allaeochelys libyca*).

With the exception of CGM 67140 and CGM 67151, the available carettochelyid shell remains from the Moghra Formation only consists of few, isolated plates of the carapace, which prevents identification of diagnostic shell features at the species level and formulation of meaningful comparisons with other carettochelyids. Taken together, the material nevertheless preserves sufficient anatomical information to allow unequivocal recognition of several features that are diagnostic of the *Allaeochelys*/*Carettochelys* lineage. In particular, the shell material resembles that of other carettochelyines by having paired nuchal processes, a midline keel, absent carapacial scutes, and a thickened pygal that anteroventrally forms a lip that is continuous with the posterior peripherals. Although we cannot exclude the presence of two species in the available sample, the shell remains are not diagnostic enough to currently support such a hypothesis. In contrast, we note that all represented individuals are similar in size and that the estimated sizes are consistent based on shell or skull remains, which supports the presence of a single, large carettochelyine taxon in the Moghra Formation. Until additional discoveries demonstrate otherwise, we attribute all Moghra shell material to *Allaeochelys meylani* sp. nov. Despite the scarce information that can be extracted from the carapacial plates, we provide some insights and comparisons with other carettochelyines wherever possible in the following section, but base our comparisons primarily on the cranial material.

Scant information can be gathered from the literature about *Allaeochelys crassesculpta* beyond the initial descriptions of Harrassowitz (1922). Though this report described all parts of the anatomy of this turtle, it only provided preliminary statements about the cranial bones and a single illustration of a reconstructed cranium in dorsal view. *Allaeochelys meylani* sp. nov. resembles *Allaeochelys crassesculpta* by having a maxilla-quadratojugal contact, a deep quadrate fossa, elongate tuberculae basioccipitale, and a completely enclosed incisura columella auris (pers. obs. on HLMD Me14997). *Allaeochelys meylani* sp. nov. can be differentiated from *Allaeochelys crassesculpta* (following Harrassowitz, 1922) by much greater size, a broader cranium, a more elongate frontal, a reduced frontal contribution to the margin of the orbit, a more elongate postorbital that has an extended medial contact with the frontal, and a more anteroposteriorly elongate pygal. *Allaeochelys meylani* sp. nov. also contrasts *Allaeochelys crassesculpta* based on the thickened cranial bones (pers. obs. SMF Me11418 and HLMD Me13721), the presence of a large fossa at the anterior third of the triturating surface (pers. obs. on HLMD Me15012 and SMF Me3004), presence of a medial process on peripheral II (pers. obs. SMF Me3018 and HLMD Me13721), and anteroposteriorly longer posterior peripherals that are nearly square (pers. obs. on HLMD Me13721 and HLMD Me15012).

Similar to the case mentioned above for *Allaeochelys crassesculpta*, few insights are available in the literature about the cranial anatomy of *Allaeochelys delheidi* despite the relative abundance of cranial remains (Alonso Santiago & Alonso Andrés, 2005; Jiménez Fuentes, 1992; Joyce, 2014). A single picture of two crania in anterior view has been published (Jimenez Fuentes, 1992), showing that *Allaeochelys delheidi* has a similar cranial shape with other carettochelyids, although the skull may be slightly flatter than that of *Allaeochelys meylani* sp. nov. and lacks the thickened cranial bones observed in the latter. Regarding the shell, *Allaeochelys meylani* sp. nov. resembles *Allaeochelys delheidi* by having square posterior peripherals but differs from it by its greater size, the presence of a medial process on peripheral II, a slightly more anteroposteriorly elongate suprapygal, and a more anteroposteriorly elongate pygal (Alonso Santiago et al., 2008; Dollo, 1886).

No anatomical comparisons are possible between *Allaeochelys meylani* sp. nov. and *Allaeochelys lignanica* (Young & Chow, 1962) as there is no overlap between the shell material reported for the two taxa. It is clear, nonetheless, that *Allaeochelys meylani* sp. nov. is much larger.

As *Allaeochelys parayrei* is mostly known by shell material (also a mandible and postcranial remains, but no cranium; see Broin, 1977; Joyce, 2014), our comparisons with *Allaeochelys meylani* sp. nov. are very limited. The shell of *Allaeochelys meylani* sp. nov. resembles that of *Allaeochelys parayrei* (following Broin, 1977) by an anteroposteriorly elongate pygal and similar proportions of the suprapygal, but differs from it by its greater size, presence of a medial process on peripheral II, slightly more elongate peripheral IV, and a midline keel on the pygal that is less extended posteriorly.

*Allaeochelys magnifica* is exclusively known by shell material and comparisons are therefore minimal for this taxon as well (Hutchison et al., 2004). The shell of *Allaeochelys meylani* sp. nov. resembles that of *Allaeochelys magnifica* by having square posterior peripherals but differs from it by its smaller size, a broader suprapygal posteriorly, a shorter midline keel on the pygal that is less extended posteriorly, and lateral margins of the pygal that are nearly parallel.

*Allaeochelys liliae* is only known by its holotype, an incomplete posterior part of the shell, which again limits the extent of comparisons (Carbot-Chanona et al., 2020). The shell of *Allaeochelys meylani* sp. nov. resembles that of *Allaeochelys liliae* by similar proportions of costals VIII and the suprapygal and a well-defined sinusoid shaped hypo-xiphiplastral suture at its lateral end but differs from it by its greater size.

*Allaeochelys meylani* sp. nov. resembles *Carettochelys niahensis* (following White et al., 2023) based on the presence of an ophthalmic nerve foramen, an anteroposteriorly elongate contact between the crista cranii of the frontal and descending process of the prefrontal, a reduced foramen interorbitale, and a maxilla-quadratojugal contact below the jugal. *Allaeochelys meylani* sp. nov. differs from *Carettochelys niahensis* based on thickened cranial bones, a narrower sulcus olfactorius, a reduced contribution of the jugal to the orbit margin, the absence of a cheek emargination, a reduced contribution of the frontal to the orbit margin, and a medial contact of the postorbital with the frontal for most of the anteroposterior length of both elements.

*Allaeochelys meylani* sp. nov. resembles *Allaeochelys libyca* (following Havlik et al., 2014; Rollot et al., 2024b) in the presence of an ophthalmic nerve foramen, a shallow fossa formed by the parietal ventromedial to the lip continuous with the processus trochlearis oticum, a dorsoventrally tall posteroventral process of the quadratojugal, the presence of a foramen oropharyngeale, a deep quadrate fossa, a well-developed enfolded ridge that covers the pterygoid fossa, a broad pterygoid fossa, and a semicircular depression on the ventral surface of the basioccipital. *Allaeochelys meylani* sp. nov. can be differentiated from *Allaeochelys libyca* based on its much greater size, a taller cranium, thickened cranial bones, a convex suture of the prefrontal with the frontal, a broader quadrate anteriorly, a narrower sulcus olfactorius, an elongate contact of the crista cranii of the frontal with the descending process of the prefrontal, a constricted and narrower anterior section of the braincase, a longer canalis caroticus internus, a larger foramen arteriomandibulare, a steeper boundary between the two articular facets of the mandibular condyle, presence of a shallow recess in the quadrate anterodorsal to the incisura columella auris, a reduced hiatus acusticus, a geniculate ganglion located more medially within the prootic, a thicker parabasisphenoid with a taller posterior contact with the basioccipital, a single external foramen for the hypoglossal nerve, and a subtle dorsal embayment of the common crus.

*Allaeochelys meylani* sp. nov. from the late Early Miocene (Burdigalian) Moghra Formation of Egypt is closest in time and space among previously named carettochelyids with *Allaeochelys libyca* from the early Middle Miocene (Langhian) Lower Maradah Formation of Libya (Havlik et al., 2014). Although it could be interpreted that the two taxa represent juvenile (*libyca*) and adult (*meylani*) forms of the same species, we note that all previously reported shell material from the Lower Maradah Formation supports the sole presence of a small taxon (Havlik et al., 2014), while all available shell material from the Moghra Formation indicates a very large taxon. So, while the two taxa are apparently close in morphology, we suspect they inhabited two distinct, only poorly connected ancient river systems that allowed allopatric speciation, much like recent *Graptemys* species along the Gulf Coast of North America (TTWG, 2021).

*Allaeochelys meylani* sp. nov. resembles the extant *Carettochelys insculpta* (following Rollot et al., 2024a; Waite, 1905; Walther, 1922) based on a depression in the roof of the orbit delineated by a ridge formed along the anteroventral surface of the frontal, a reduced jugal contribution to the orbit margin, a reduced contribution of the frontal to the orbit margin, a maxilla-quadratojugal contact below the jugal, presence of an anterior foramen for the exit of the canalis alveolaris superior located along the ventrolateral margin of the external nares, presence of a fossa along the anterior aspect of the triturating surface, a deep pterygoid fossa, presence of a foramen oropharyngeale, a completely enclosed fenestra perilymphatica, a semicircular depression on the ventral surface of the basioccipital, square posterior peripherals, similar proportions of the suprapygal, and an anteroposteriorly elongate pygal. *Allaeochelys meylani* sp. nov. differs from *Carettochelys insculpta* by the presence of an ophthalmic nerve foramen, thickened cranial bones, a convex suture of the prefrontal with the frontal, a medial contact of the postorbital with the frontal for most of the anteroposterior length of both elements, a broader upper temporal emargination that fully exposes the prootic in dorsal view, a narrower sulcus olfactorius, a reduced foramen interorbitale, the presence of two shallow fossae ventral to the lip that is continuous with the processus trochlearis oticum, a mediolaterally expanded bony wall posterior to the orbit formed by the jugal and postorbital, a dorsoventrally taller posteroventral process of the quadratojugal, a contribution of the maxilla to the foramen palatinum posterius, a broader pterygoid fossa, a longer canalis caroticus internus, a large foramen arteriomandibulare, a steeper boundary between the two articular facets of the mandibular condyle, a deeper quadrate fossa, a fenestra ovalis completely enclosed by the prootic and opisthotic, a reduced hiatus acusticus, a geniculate ganglion located more medially within the prootic, a thicker parabasisphenoid with a taller posterior contact with the basioccipital, a single external foramen for the hypoglossal nerve, a subtle dorsal embayment of the common crus, the presence of a medial process on peripheral II, and nearly parallel lateral margins of the pygal.

### Comments on the carettochelyid remains reported by Dacqué (1912)

Dacqué (1912: p. 322, plate 2 Figs. 1, 2) published and figured a now lost fragment from the Burdigalian of Wadi Faregh, Egypt that he identified as a plastral element that possibly represented a new species of the trionychid *Cyclanorbis*, but the specimen was later assigned to aff. *Allaeochelys* sp. by de Lapparent de Broin (2000). The illustrations provided by Dacqué (1912: plate 2 Figs. 1, 2) clearly show the bone surface ornamentation that is typical for carettochelyids, as well as the absence of scute sulci, which together allow for the referral of the specimen to the *Allaeochelys*/*Carettochelys* lineage, consistent with de Lapparent de Broin (2000). However, it is apparent that this specimen is not part of the plastron but rather consists of a partial nuchal and left peripheral I based on the shape of the bones, an intact margin along one entire side of the specimen that represents the anterior carapace margin, and the presence of processes on the ventromedial surface of the element that correspond to paired nuchal processes. The size of the specimen is not given by Dacqué (1912). We assume that his illustrations are to scale since scale bars are provided on other plates for specimens that are figured smaller than actual size. Following this, the left peripheral I is estimated to have a mediolateral length of approximately 8 cm (measurement taken at a right angle from the lateral suture with left peripheral II towards the medial suture with the nuchal), which is about the same size as the peripherals and pygal reported here as 6−8 cm. Also, the shape of the preserved portions of the nuchal and left peripheral I is identical to that of CGM 67151. As the material from Dacqué (1912) can be referred to *Allaeochelys*, has the same age as the material from Moghra that we report herein, has the same size as the Moghra material, and also appears to be identical to CGM 67151, we choose to refer the nuchal and left peripheral I from Wadi Faregh to *Allaeochelys meylani* sp. nov.

### Alpha taxonomy—shell material of *Trionyx* sp.

The fossil record of pan-trionychids from Afro-Arabia is scarce and only three taxa are currently recognized as valid from this continent, all of which are cyclanorbines from the Neogene of Kenya: *Cyclanorbis turkanensis* from the early Pliocene (Meylan et al., 1990), *Cycloderma victoriae* from the Early Miocene (Andrews, 1914), and *Cycloderma debroinae* from the early Pliocene (Meylan et al., 1990). Additional sporadic discoveries have been reported from across Arabia and North and East Africa but comprise only fragmentary material that can only be identified to the level of *Pan-Trionychidae* indet. (see Georgalis & Joyce, 2017 and references therein; Georgalis, 2021), including the type of *Trionyx senckenbergianus*, which is from Moghra (Reinach, 1903). Globally speaking, the Miocene fossil record of pan-trionychids is also scarce, with only three additional valid taxa being recognized. These are “*Trionyx*” *miocaenus* from the late Middle Miocene of North America (Matthew, 1924), *Rafetus bohemicus* from the late Early Miocene of Czechia (Chroust et al., 2023; Liebus, 1930), and *Trionyx vindobonensis* from the late Middle Miocene of Europe (Peters, 1855), but fossil calibrated molecular trees suggest that sampling is very incomplete, as most extant taxa have stem lineages that extend throughout the Miocene (e.g., Thomson et al., 2021).

While trionychids are absent from Europe today, four lineages are present in Afro-Arabia, in particular the cyclanorbines *Cyclanorbis* spp. and *Cycloderma* spp., as well as the trionychines *Trionyx triunguis* (Forskål, 1775) and *Rafetus euphraticus* (Daudin, 1801; TTWG, 2021). As the Neogene fossil record does not provide any positive evidence for other lineages in the region (Georgalis & Joyce, 2017) and since our material is highly consistent with a referral to *Trionyx* sp., we focus our comparisons on these clades and disregard other lineages of pan-trionychids (e.g., *Plastomenidae*, *Amydini*, *Apalone* spp.). However, as the carapace and plastral remains from Moghra were collected from different localities, we provide separate comparisons and rationales for their taxonomic attribution to avoid discussing a chimaera.

The carapace of DPC 7789 can readily be distinguished from that of cyclanorbines by the absence of a preneural, a relatively small costal VIII, no underlap of costal I by the nuchal processes, a broader and rounded margin of the posterior half of the carapace, and broad rib ends that extend past the lateral costal margins (Georgalis & Joyce, 2017; Joyce, 2025). DPC 7789 differs most apparently from *Rafetus* spp. by the presence of an anteroposteriorly shorter, a less rounded nuchal, larger costals VIII, and a nuchal-costal I suture that is strongly curved anteriorly. No major difference can be spotted between DPC 7789 and *Trionyx* spp. (Joyce, 2025), with the exception of the nuchal-costal I suture, which is somewhat more strongly curved towards the anterior in DPC 7789 than in *Trionyx triunguis* (although variation is apparent for this feature in the latter).

The hyoplastron DPC 4466 can be distinguished from that of cyclanorbines by not being fused to the hypoplastron and by having a relatively expanded comb of medial hyoplastral processes, and from that of *Rafetus* spp. by having a much more developed hyoplastral callosity. No particular difference can be identified between DPC 4466 and the hyoplastron of *Trionyx* spp. (Joyce, 2025).

The hypoplastra DPC 4122 and DPC 6436 can be distinguished from that of cyclanorbines by lacking fusion with the hyoplastron and from *Rafetus* spp. by the presence of an expanded hypoplastral callosity (Georgalis & Joyce, 2017; Vitek & Joyce, 2015; Joyce, 2025). DPC 6436 can furthermore be differentiated from *Rafetus* spp. but resembles *Trionyx* spp. by the presence of an expanded medial hypoplastral comb, which among pan-trionychids is a synapomorphy of *Chitrini*, the clade that unites *Chitra* spp., *Pelochelys* spp., and *Trionyx* spp. (sensu Joyce, 2025).

Our comparisons highlight that the trionychid material reported herein from the Moghra Formation is more similar to *Trionyx* spp. than any cyclanorbine and *Rafetus* spp. However, two European fossil *Trionyx* species are currently recognized from the Neogene that may plausibly represent a single phylogenetic lineage: *Trionyx vindobonensis* from the Miocene (Georgalis & Joyce, 2017; Peters, 1855) and *Trionyx pliocenicus* from the Pliocene (Fucini, 1912; Georgalis & Joyce, 2017). Considered together, the Moghra specimens greatly resemble these *Trionyx* species by having a similar carapace outline, typically seven elongate neurals, a deep nuchal, relatively small costal VIII, reduced costal rib VIII, similar shape and proportions of the hyoplastral callosity, and the presence of an expanded medial hypoplastral comb. The two fossil taxa are only distinguished from one another and the extant *Trionyx triunguis* by nuances in the morphology of the plastron (Georgalis & Joyce, 2017) and these characteristics should be reevaluated in light of recent insights into ontogenetic changes in trionychids in general (Joyce, 2025). We are therefore confident in our attribution of the Moghra material to the *Trionyx* lineage, but suggest that additional plastral material in combination with a review of plastral characters in the *Trionyx* lineage are needed to assign the material to a particular species.

## Data Availability

The original set of μCT scans and 3D models of bones of DPC 7742 are available at MorphoSource (https://www.morphosource.org/projects/000694201).
